# Meta-analysis of GWA studies provides new insights on the genetic architecture of skin pigmentation in recently admixed populations

**DOI:** 10.1186/s12863-019-0765-5

**Published:** 2019-07-17

**Authors:** Frida Lona-Durazo, Natalia Hernandez-Pacheco, Shaohua Fan, Tongwu Zhang, Jiyeon Choi, Michael A. Kovacs, Stacie K. Loftus, Phuong Le, Melissa Edwards, Cesar A. Fortes-Lima, Celeste Eng, Scott Huntsman, Donglei Hu, Enrique Javier Gómez-Cabezas, Lilia Caridad Marín-Padrón, Jonas Grauholm, Ole Mors, Esteban G. Burchard, Heather L. Norton, William J. Pavan, Kevin M. Brown, Sarah Tishkoff, Maria Pino-Yanes, Sandra Beleza, Beatriz Marcheco-Teruel, Esteban J. Parra

**Affiliations:** 1 Department of Anthropology, University of Toronto at Mississauga, Health Sciences Complex, room 352, Mississauga, Ontario L5L 1C6 Canada; 2 Research Unit, Hospital Universitario N.S. de Candelaria, Universidad de La Laguna, Santa Cruz de Tenerife, Spain; 3 Genomics and Health Group, Department of Biochemistry, Microbiology, Cell Biology and Genetics, Universidad de La Laguna, La Laguna, Tenerife, Spain; 4 Department of Genetics, Perelman School of Medicine, University of Pennsylvania, Philadelphia, USA; 5 Laboratory of Translational Genomics, Division of Cancer Epidemiology and Genetics, National Cancer Institute, National Institutes of Health, Bethesda, USA; 6 Genetic Disease Research Branch, National Human Genome Research Institute, National Institutes of Health, Bethesda, USA; 7 Evolutionary Anthropology Team, Laboratory Eco-Anthropology and Ethno-Biology UMR7206, CNRS-MNHN-University Paris Diderot, Musée de l’Homme, Paris, France; 8 Department of Organismal Biology, Evolutionary Biology Centre, Uppsala University, Uppsala, Sweden; 9 Department of Bioengineering and Therapeutic Sciences, University of California, San Francisco, CA USA; 10 Centre for Sociological and Psychological Research, Havana, Cuba; 11 National Centre of Medical Genetics, Medical University of Havana, La Habana, Cuba; 12 Department for Congenital Disorders, Statens Serum Institut, Copenhagen, Denmark; 13 Translational Neuropsychiatry Unit, Department of Clinical Medicine, Aarhus University, Aarhus, Denmark; 14 The Lundbeck Foundation Initiative for Integrative Psychiatric Research, Aarhus University, Aarhus, Denmark; 15 Psychiatric Department, Aarhus University Hospital, Aarhus, Denmark; 16 Department of Anthropology, University of Cincinnati, Cincinnati, USA; 17 Department of Biology, School of Arts and Sciences, University of Pennsylvania, Philadelphia, PA USA; 18 CIBER de Enfermedades Respiratorias, Instituto de Salud Carlos III, Madrid, Spain; 19 Instituto de Tecnologías Biomédicas (ITB), Universidad de La Laguna, Santa Cruz de Tenerife, Spain; 20 Department of Genetics and Genome Biology, College of Life Sciences, University of Leicester, Leicester, UK

**Keywords:** Skin pigmentation, Genome-wide association study, Admixed populations, Meta-analysis, Complex trait, Gene expression, Haplotype

## Abstract

**Background:**

Association studies in recently admixed populations are extremely useful to identify the genetic architecture of pigmentation, due to their high genotypic and phenotypic variation. However, to date only four Genome-Wide Association Studies (GWAS) have been carried out in these populations.

**Results:**

We present a GWAS of skin pigmentation in an admixed sample from Cuba (*N* = 762). Additionally, we conducted a meta-analysis including the Cuban sample, and admixed samples from Cape Verde, Puerto Rico and African-Americans from San Francisco. This meta-analysis is one of the largest efforts so far to characterize the genetic basis of skin pigmentation in admixed populations (*N* = 2,104). We identified five genome-wide significant regions in the meta-analysis, and explored if the markers observed in these regions are associated with the expression of relevant pigmentary genes in human melanocyte cultures. In three of the regions identified in the meta-analysis (*SLC24A5*, *SLC45A2,* and *GRM5/TYR*), the association seems to be driven by non-synonymous variants (rs1426654, rs16891982, and rs1042602, respectively). The rs16891982 polymorphism is strongly associated with the expression of the *SLC45A2* gene. In the *GRM5/TYR* region, in addition to the rs1042602 non-synonymous SNP located on the *TYR* gene, variants located in the nearby *GRM5* gene have an independent effect on pigmentation, possibly through regulation of gene expression of the *TYR* gene. We also replicated an association recently described near the *MFSD12* gene on chromosome 19 (lead variant rs112332856). Additionally, our analyses support the presence of multiple signals in the *OCA2/HERC2/APBA2* region on chromosome 15. A clear causal candidate is the *HERC2* intronic variant rs12913832, which has a profound influence on *OCA2* expression. This variant has pleiotropic effects on eye, hair, and skin pigmentation. However, conditional and haplotype-based analyses indicate the presence of other variants with independent effects on melanin levels in *OCA2* and *APBA2*. Finally, a follow-up of genome-wide signals identified in a recent GWAS for tanning response indicates that there is a substantial overlap in the genetic factors influencing skin pigmentation and tanning response.

**Conclusions:**

Our meta-analysis of skin pigmentation GWAS in recently admixed populations provides new insights about the genetic architecture of this complex trait.

**Electronic supplementary material:**

The online version of this article (10.1186/s12863-019-0765-5) contains supplementary material, which is available to authorized users.

## Background

Constitutive human skin pigmentation (i.e. pigmentation of unexposed skin) is one of the most diverse phenotypes among worldwide populations. Previous work indicates that there has been selective pressure upon this phenotype at a global scale, with higher melanin levels favored in equatorial and tropical regions due to its protective effects against the harmful effects of ultraviolet radiation, and lighter skin pigmentation in higher latitudes, possibly because it facilitates cutaneous synthesis of vitamin D [1, 2].

Association studies in recently admixed populations can be extremely useful to identify the genetic architecture of pigmentation, due to their wide range of genotypic and phenotypic variation, and the increased statistical power to detect associations for genetic markers that have extreme frequencies in the parental population groups [3]. For instance, the identification of the genomic regions with the strongest effect reported on skin pigmentation (*SLC24A5* and *SLC45A2*) was originally done in admixed individuals [4, 5]. Unfortunately, to date only four genome-wide pigmentation studies have been conducted in recently admixed populations [6–9]. Similarly, in spite of the fact that there is substantial variation in the skin pigmentation spectrum within the African continent, to date only two GWAS have been conducted in African populations [10, 11].

Here, we present the results of a GWAS of skin pigmentation in an admixed sample from Cuba [12]. Additionally, to increase statistical power, we carried out a meta-analysis including available genome-wide single-nucleotide polymorphism (SNP) data of four recently admixed samples: the Cuban sample, the Cape Verde sample [6], and the Puerto Rican and African American samples [8]. These samples are characterized by different patterns of admixture between European and West African populations. The overall sample includes 2,104 individuals. To our knowledge, this is one of the largest meta-analysis conducted so far for skin pigmentation in admixed populations. We also followed-up the genome-wide signals that have been described in previous GWAS in recently admixed samples and in samples from the African continent [10, 11].

## Results

### GWAS of skin pigmentation in the Cuban sample

The distribution of M-index in the Cuban sample has a wide range of M-index values, from the low 20s to higher melanin content than 80 (Additional file 1: Figure S1). On average, M-index was 39.88 (SD = 10.01). The top variant identified in the GWAS is rs35397 (*p* = 9.49 × 10^− 13^), located within the gene *SLC45A2* on chromosome 5 (Additional file 1: Figure S2). Variants located within the gene *SLC24A5* on chromosome 15 were also close to genome-wide significance, with the known non-synonymous rs1426654 variant being the top SNP in this region (*p* = 3.42 × 10^− 7^). A QQ plot of the GWAS *p*-values shows the observed vs. expected distribution, with deviations at the extreme tail of the distribution (λGC = 1.046; Additional file 1: Figure S3). Detailed information about the genome-wide (*p* < 10^− 8^) and suggestive signals (*p* < 10^− 5^) identified in the analysis is available in the Additional file 3.

### Meta-analysis of association results in four admixed samples

We combined the summary statistics of the Cuban sample with three available datasets from Cape Verde [6], African Americans and Puerto Ricans [8]. All of the individual GWAS were carried out using the same analytical strategy and took into account population structure (i.e. individual admixture proportions, for more detailed information see Methods section). Prior to performing the meta-analysis, the standard errors of the beta coefficients were corrected based on the estimated lambda values. The ancestry estimates for each of the datasets are summarized in Table 1. There is a significant correlation of African individual proportions and melanin index in each of the samples, but there are clear differences in the percentage of skin pigmentation variation explained by ancestry in the Cuban (64.5%), Cape Verdean (43.6%), African-American (21.6%), and Puerto Rican (18.2%) sample (Additional file 1: Figures S4-S7).

**Table 1:** Average estimates of African, European, Native American and East Asian ancestry proportions in datasets

Sample	Cuba^a^	SAGE II^b^	GALA II^b^	Cape Verde^c^
Size	762	373	285	684
African (%)	20.3	80.9	22.8	58
European (%)	71.1	19.10	66.8	42
Native American (%)	6.8	NA	10.40	NA
East Asian (%)	1.8	NA	NA	NA

*NA* Not applicable

Data source:

^a^Fortes-Lima et al. 2018 [12]

^b^Hernandez-Pacheco et al. 2017 [8]

^c^Beleza et al. 2013 [6]

**Fig. 1:**
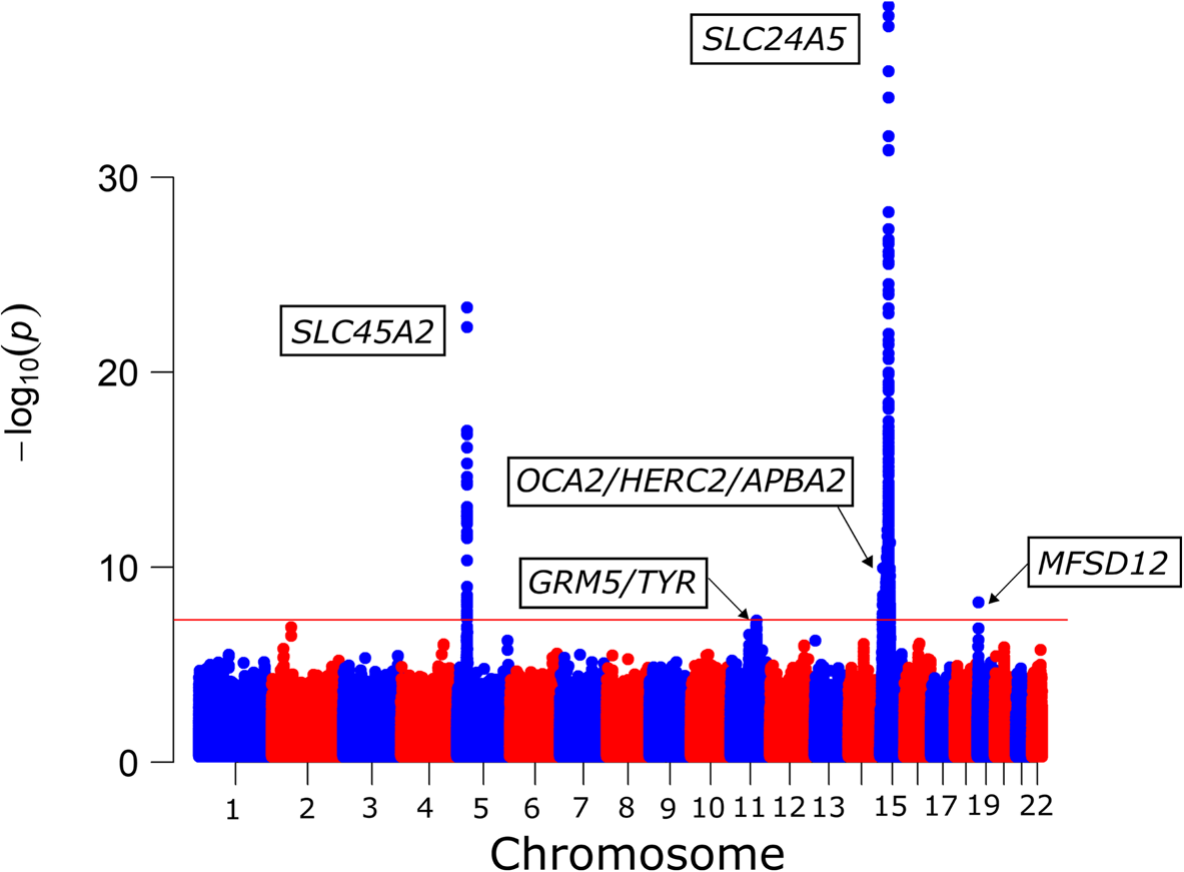
Manhattan plot depicting the results of the meta-analysis of four African admixed samples. The red horizontal indicates the genome-wide significance threshold (*p* = 5 × 10^−8^)

The Manhattan plot depicting the results of the meta-analysis is shown in Fig. 1, and the QQ plot is depicted in Additional file 1: Figure S8. As expected, both rs1426654 (*SLC24A5*) and rs35397 (*SLC45A2*) show very strong effects in the meta-analysis (rs1426654, *p* = 6.32 × 10^− 39^, and rs35397, *p* = 1.98 × 10^− 24^, respectively). The SNP with the second lowest *p*-value (*p* = 2.13 × 10^− 23^) in the *SLC45A2* region is the non-synonymous variant rs16891982. Additional file 2: Table S1 provides information about all the genome-wide signals identified in the meta-analysis, including *p*-values, estimates of between-study heterogeneity (Cochran’s Q and corresponding *p*-value and I^2^ value), *p*-values and M-values of the individual studies, and allele frequencies of the tested alleles in the study populations, as well as African and European populations.

**Fig. 2:**
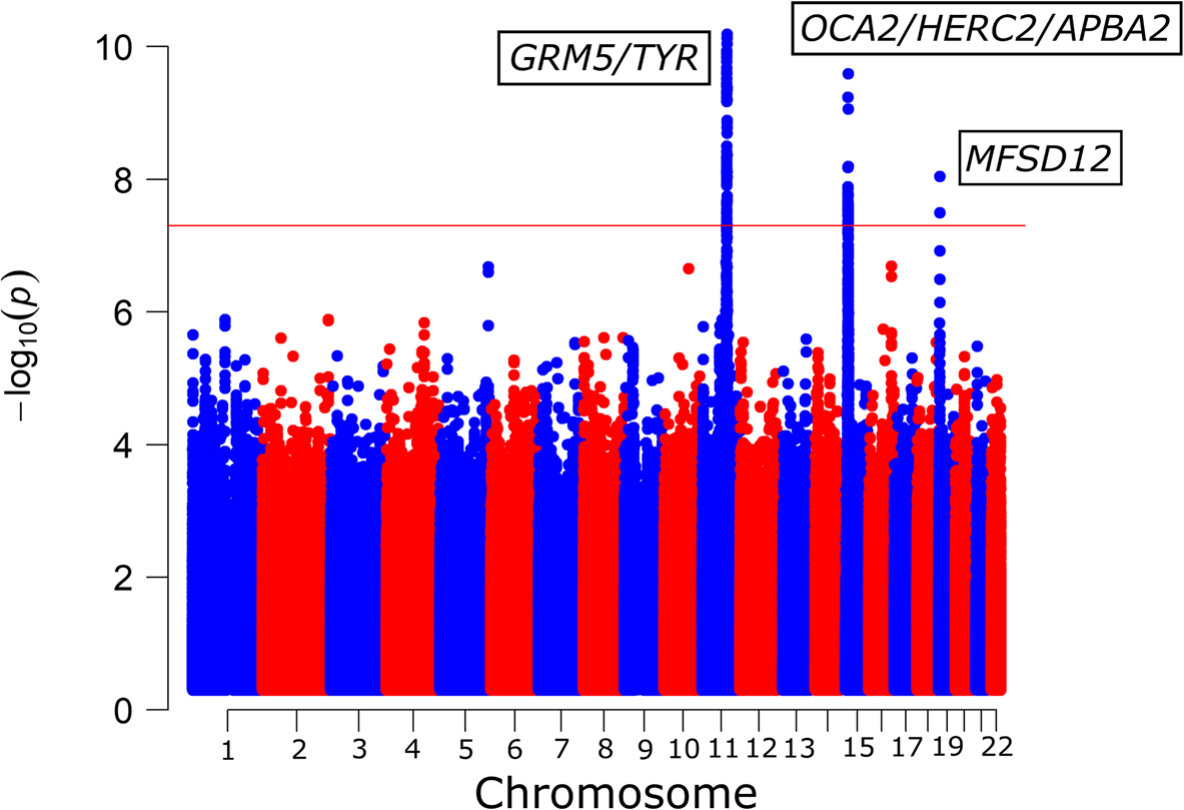
Manhattan plot depicting the results of the conditional meta-analysis (conditioned for the effects of the two major SNPs: rs1426654 in *SLC24A5* and rs35397 in *SLC45A2*) of four African admixed samples. The red horizontal indicates the genome-wide significance threshold (*p* = 5 × 10^− 8^)

Given the very strong effects observed in the *SLC24A5* and *SLC45A2* regions, the meta-analysis was repeated after controlling for the polymorphisms rs1426654 and rs35397 in each study. Figure 2 depicts the Manhattan plot corresponding to this analysis, and the QQ plot (λ_GC_ = 1.016) is presented on Additional file 1: Figure S9. Further details about the genome-wide signals identified in this analysis are provided in Table 2 (meta-analysis results) and Additional file 2: Table S2 (meta-analysis results, and *p*-values and allele frequencies in each sample). The results with and without controlling for *SLC24A5* and *SLC45A2* are very consistent. Detailed information of the genome-wide and suggestive signals identified in the main meta-analysis and the meta-analysis controlling for *SLC24A5* and *SLC45A2* are available in the Additional file 3.

**Table 2:** Top genome-wide significant SNPs in conditional meta-analysis and on each independent study

SNP	Chr	Position (GRCg37/hg19)	EA/ NEA	Genes^a^	Fixed Effects Model^b^			Random Effects Model^c^ *P*-value	Cochran’s Q		I^2^
					*P*-value	Beta	SD		Q	*P*-value	
rs10160510	11	88614324	T/A	*GRM5*	3.36E-10	−0.248	0.039	7.54E-11	11.078	0.011	72.920
rs3098576	15	27858408	T/C	*(GABRG3/OCA2)*	8.90E-09	0.182	0.032	1.30E-08	2.883	0.410	0.000
rs1448484	15	28283441	G/A	*OCA2*	3.73E-10	0.213	0.034	5.83E-10	2.237	0.525	0.000
rs1667392	15	28533565	C/G	*HERC2*	4.64E-09	−0.268	0.046	6.65E-09	1.048	0.306	4.580
rs36194177	15	29118784	A/G	*(APBA2)*	4.95E-10	0.229	0.037	2.58E-10	9.281	0.026	67.676
rs2636060	15	29425936	A/G	*FAM189A1*	5.71E-10	−0.231	0.037	8.79E-10	3.433	0.330	12.611
rs10416746	19	3563982	A/G	*(MFSD12)*	1.01E-09	0.309	0.051	1.55E-09	3.947	0.267	24.002

The table presents the results of the meta-analysis after conditioning for *SLC24A5* rs1426654 and *SLC45A2* rs35397, which had very strong effects in the original meta-analysis (*p* = 6.32 × 10^− 39^, and rs35397, *p* = 1.98 × 10^− 24^, respectively)

*Chr* Chromosome, *EA* Effect allele, *NEA* Non-effect allele, *SD* Standard deviation, *M* Posterior probability of an existent effect on each study, *I*^*2*^ Heterogeneity statistic, *NA* Marker not genotyped

^a^For variants located in intergenic regions, nearby genes are indicated in parenthesis

^b^Fixed effects model, as computed in Metasoft

^c^Han and Eskin’s Random Effects Model

In addition to *SLC24A5* and *SLC45A2*, we identified three regions surpassing the genome-wide significance threshold (Table 2). The first region includes multiple genome-wide significant markers overlapping with the *GRM5* and *TYR* genes on chromosome 11. The most significant marker, rs10160510 (*p* = 3.36 × 10^− 10^), is located in an intronic region of the *GRM5* gene. This marker shows evidence of effect size heterogeneity in the meta-analysis (Q *p*-value = 0.011). The second signal corresponds to an intergenic region between the genes *MFSD12* and *HMG20B* on chromosome 19 and contains two genome-wide significant markers in close proximity to each other (~ 2 kb), the most significant SNP being rs10416746 (*p* = 1.55 × 10^− 9^). The third region comprises a large number of genome-wide significant variants on chromosome 15 spanning more than 1.6 megabases (from 27.8 Mb to 29.5 Mb in the GRCh37 human assembly). This region includes the well-known *OCA2/HERC2* pigmentation genes, as well as the *APBA2* gene previously reported by Beleza et al. [6]. We provide additional analyses of this region below.

### Linkage disequilibrium, conditional and haplotype analyses of *OCA2/HERC2/APBA2* region

There are at least four clusters of genome-wide significant variants, each of them separated by more than 250 Kb, in the 27.8–29.5 Mb interval on chromosome 15. We carried out a detailed analysis of linkage disequilibrium (LD) patterns between the genome-wide signals observed in the Cuban and Cape Verde samples, as well as the PUR and ASW samples. Additional file 1: Figures S10-S17 show in graphical format the r^2^ and D’ values between the genome-wide significant SNPs. Additionally, Additional file 1: Figures S18-S27 show the regional plots corresponding to this region, highlighting the LD patterns of the lead SNPs in each cluster in African and European populations.

The first cluster includes multiple genome-wide significant markers in strong LD upstream of the gene *OCA2.* The top SNP in this cluster is rs3098576 (*p* = 8.9 × 10^− 9^). Based on the r^2^ and D’ estimates, the markers in this cluster are not in strong LD (D’ < 0.5) with any of the lead SNPs located in the other clusters, including a cluster located approximately 400 Kb apart within the gene *OCA2*.

The second cluster includes markers located within the *OCA2/HERC2* genes, from 28.28 Mb to 28.54 Mb. The most significant marker within *OCA2* is rs1448484 (*p* = 3.73 × 10^− 10^), located in an intronic region of the gene. In *HERC2*, the top marker is rs1667392 (*p* = 4.64 × 10^− 9^). *OCA2* rs1448484 and *HERC2* rs1667392 are separated by approximately 250 Kb.

The third cluster corresponds to SNPs close to or within the gene *APBA2*. This cluster is located more than 550 Kb away from the *OCA2/HERC2* cluster. The top marker in this region is rs36194177 (*p* = 4.95 × 10^− 10^). For this marker there is evidence of effect size heterogeneity in the meta-analysis (Q *p-*value = 0.026).

The fourth cluster is located within the gene *FAM189A1*, which is located approximately 270 Kb away from the *APBA2* cluster. The top SNP in this region is rs2636060, (*p* = 5.71 × 10^− 10^).

As shown in Additional file 1: Figures S10-S17, although the *r*^2^ values between the markers located in different clusters are lower than 0.5 in all the studied populations, the D’ values, which are not as influenced by allele frequencies, indicate that the markers located in clusters 2–4 are in linkage disequilibrium (D’ > 0.8 in many inter-marker comparisons). For this reason, we also carried out conditional tests and association analyses at the haplotype level in the samples of Cape Verde and Cuba.

We first performed association tests conditioning for each of the lead signals in this region: rs1448484 (*OCA2*), rs1667392 (*HERC2*), rs36194177 (*APBA2*) and rs2636060 (*FAM189A1)*. The results of these analyses are reported in Additional file 2: Table S3. As expected given the patterns of LD observed in the Cape Verde and Cuban samples, conditioning for the lead SNPs in the *OCA2/HERC2/APBA2* region decreases the beta estimates and the *p*-values of the other markers. However, in the Cape Verde sample all of the markers remain nominally significant after conditioning, except for rs1448484 after conditioning for rs2636060.

We also carried out association tests based on haplotypes for rs1448484, rs1667392, rs36194177, and rs2636060*.* The results of the haplotype association tests for the Cape Verde and Cuban sample are shown in Additional file 2: Tables S4 and S5, respectively. The beta values report the effect sizes estimated for each haplotype, using the haplotype GGAG as the reference. This haplotype contains the alleles that are common in African populations and are in all cases associated with higher melanin index values in the meta-analysis.

In the Cape Verde sample, the omnibus haplotype test is significant (*p* = 1.38 × 10^− 6^) and the amount of skin pigmentation variation explained by the model is 6.3%. The haplotype with the strongest effect is the ACGA haplotype, which includes all the alleles common in European populations (beta = − 0.466). Other haplotypes associated with lower melanin levels are GGGA (beta = − 0.326), AGGA (beta = − 0.268) and AGGG (beta = − 0.229). In the sample from Cuba, the omnibus test is also significant (*p* = 0.0131) and the haplotype model explains 2.73% of the skin pigmentation variation. In this sample, the results are quite consistent with those observed in the Cape Verde sample. The haplotypes showing the strongest effects on pigmentation are AGGG (beta = − 0.330), ACGA (beta = − 0.322) and GGGA (beta = − 0.202).

In addition to the omnibus tests, we also carried out tests controlling for the haplotypes with the strongest effects, to explore if a single haplotype can explain the observed effects. In the sample of Cape Verde, after controlling for the haplotype ACGA, which has all the alleles common in European populations, there is still a marginal association (*p* = 0.0182), as is the case after controlling for ACGA and GGGA (*p* = 0.0204). Controlling for ACGA, GGGA and AGGA, the model is not significant. In the Cuban sample, after controlling for ACGA, the results are not significant.

### Association of top meta-analysis variants with gene expression data

We evaluated the potential association of the genome-wide significant signals identified in the meta-analysis with gene expression, using transcriptome and genotype data from primary cultures of human melanocytes isolated from foreskin of more than 100 individuals of diverse ancestries [13]. Table 3 shows the strength of association of the meta-analysis top signals with the expression of key pigmentation genes (*SLC45A2*, *TYR*, *OCA2* and *SLC24A5*), and for comparative purposes, also reports the variants in each region with the strongest association to gene expression, and their respective *p*-values in the meta-analysis. The two markers on chromosome 5 showing the strongest associations with melanin levels in the meta-analysis are among the variants with the strongest effects on *SLC45A2* expression (nominal *p*-values for *SLC45A2* expression, rs35397, *p* = 3.95 × 10^− 5^, and rs16891982, *p* = 4.78 × 10^− 7^). In the *OCA2/HERC2/APAB2* region, five of the genome-wide signals were associated with expression of the key pigmentary gene *OCA2.* Four of these variants are located in the *HERC2* gene (nominal *p*-values for *OCA2* expression, rs12913832, *p* = 3.14 × 10^− 23^; rs1129038, *p* = 1.24 × 10^− 22^; rs6497263, *p* = 6 × 10^− 3^; and rs73377768, *p* = 6.6 × 10^− 3^). The variant rs1448484, which is located in the *OCA2* gene, was also nominally associated with *OCA2* expression (*p* = 0.022). It is important to note that several of the genome-wide signals identified in the *OCA2/HERC2/APAB2* region, including some variants within or near the *HERC2, APAB2* and *FAM189A1* genes, were not present in the expression quantitative trait loci (eQTL) dataset, due to poor imputation quality or low allele frequency (minor allele frequency (MAF) < 0.01), so it was not possible to evaluate if these variants are associated with gene expression. Similarly, the two genome-wide significant variants identified in the chr19 region were not present in this dataset.

**Table 3:** Association of the meta-analysis top signals with the expression of key pigmentation genes (*SLC45A2*, *TYR*, *OCA2* and *SLC24A5*)

SNP	EA	Chr	Position (GRCg37/hg19)	Note	*P*-value (meta-FE model)	Melanin	Gene	*P*-value (gene expression)	Expression
Chromosome 5 region (*SLC45A2*) lead SNPs (meta-analysis)									
rs35397	T	5	33951116		1.98E-24	↓	*SLC45A2*	3.95E-05	↑
**rs16891982**	**G**	**5**	**33951693**	**Non-synonymous**	**2.13E-23**	**↓**	***SLC45A2***	**4.78E-07**	↑
Chromosome 5 region (*SLC45A2*) lead eQTLs									
rs35407	G	5	33946571		3.08E-15	↓	*SLC45A2*	2.11E-07	↑
**rs16891982**	**G**	**5**	**33951693**	**Non-synonymous**	**2.13E-23**	**↓**	***SLC45A2***	**4.78E-07**	↑
Chromosome 11 region (*GRM5/TYR*) lead SNPs (meta-analysis)									
rs10160510	T	11	88614324		3.36E-10	↓	*TYR*	3.09E-03	↓
rs10437581	A	11	88616875		4.89E-10	↓	*TYR*	2.26E-03	↓
rs1042602	A	11	88911696	Non-synonymous	9.20E-10	↓	*TYR*	2.73E-02	↓
rs12801588	G	11	88548272		9.62E-10	↓	*TYR*	1.98E-03	↓
Chromosome 11 region (*GRM5/TYR*) lead eQTLs									
rs28437494	G	11	88868611		0.102	↓	*TYR*	1.78E-06	↓
rs4121729	G	11	88863140		0.038	↓	*TYR*	1.88E-06	↓
rs7925346	T	11	88860927		0.035	↓	*TYR*	1.91E-06	↓
Chromosome 15 region (*OCA2/HERC2/APBA2*) lead SNPs (meta-analysis)									
rs3098576	C	15	27858408		8.90E-09	↓	*OCA2*	0.468	↓
rs1448484	A	15	28283441		3.73E-10	↓	*OCA2*	0.022	↓
**rs12913832**	**G**	**15**	**28365618**		**3.18E-08**	**↓**	***OCA2***	**3.14E-23**	**↓**
rs1667392	C	15	28533565		4.64E-09	↓	*OCA2*	N/A	N/A
rs36194177	G	15	29118784		4.95E-10	↓	*OCA2*	N/A	N/A
rs2636060	A	15	29425936		5.71E-10	↓	*OCA2*	N/A	N/A
Chromosome 15 region (*OCA2/HERC2/APBA2*) lead eQTLs									
**rs12913832**	**G**	**15**	**28365618**		**3.18E-08**	**↓**	***OCA2***	**3.14E-23**	↓
rs1129038	T	15	28356859		3.18E-08	↓	*OCA2*	1.24E-22	↓
Chromosome 15 region (*SLC24A5*) lead SNPs (meta-analysis)									
rs1426654	A	15	48426484	Non-synonymous	6.32E-39	↓	*SLC24A5*	0.218	↓
rs2470102	A	15	48433494		2.19E-38	↓	*SLC24A5*	0.166	↓
Chromosome 15 region (*SLC24A5*) lead eQTLs									
N/A (no variants had nominal *p*-values < 0.01 in this region)									
Chromosome 19 region (*MFSD12*) lead SNPs (meta-analysis)									
rs10416746	G	19	3563982		6.20E-09	↓	*MFSD12*	N/A	N/A
rs112332856	T	19	3565599		2.31E-08	↓	*MFSD12*	N/A	N/A
Chromosome 15 region (*MFSD12*) lead eQTLs									
rs80204200	T	19	3529723		0.033761	↓	*MFSD12*	6.34E-04	↑
rs7256261	A	19	3541615		4.88E-04	↓	*MFSD12*	1.64E-03	↑

For comparative purposes, the table also reports the variants in each region with the strongest association to gene expression, and their respective *p*-values in the meta-analysis. The variants that appear as top signals in the meta-analysis and in the eQTL analysis are indicated in bold

*EA* Effect allele, *Chr* Chromosome, *FE model* Fixed effects model

In the *GRM5/TYR* region on chromosome 11, multiple genome-wide variants located within the *GRM5* gene (e.g. rs12801588, rs10160510 and rs10437581) are nominally associated with the expression of the *TYR* gene (Table 3). However, the variants identified to have the strongest association with the *TYR* gene in the eQTL study (rs28437494, rs4121729 and rs7925346), which are located more than 200 Kb apart from the aforementioned *GRM5* variants, are not nominally significant in the meta-analysis. Regarding the *SLC24A5* region, none of the genome-wide signals observed in the meta-analysis for this region were strongly associated with expression of the *SLC24A5* gene (all *p*-values were higher than 0.01 in this region). Of note, the non-synonymous variant rs1426654 was present in the imputed dataset with a frequency of 12% and high imputation confidence (*r*^2^ = 0.99948), but it was not associated with expression of *SLC24A5* (*p* = 0.218).

### Replication of lead signals of meta-analysis in East African sample

We replicated the lead SNPs identified in the meta-analysis in an East African sample (Table 4) [10]. The direction of effects is concordant for all the polymorphisms, except for rs3098576, which is located upstream of *OCA2* on chromosome 15. The markers rs1667392 (*HERC2*) and rs36194177 (*APBA2*) were not present in the imputed dataset. The polymorphism rs1426654 in *SLC24A5* is also genome-wide significant in the East African sample (*p* = 6.89 × 10^− 61^), and two additional markers are nominally significant (rs1448484 in *OCA2*, *p* = 4.43 × 10^− 4^ and rs10416746 in *MFSD12*, *p* = 1.53 × 10^− 4^). The results of the follow-up for all the genome-wide signals are presented in Additional file 2: Table S6, providing additional relevant information. The second most significant SNP observed in the meta-analysis in the *MFSD12* region, rs112332856, reaches genome-wide significance in the East African sample (*p* = 1.15 × 10^− 15^). Additionally, many of the genome-wide signals identified in the *GRM5* gene in the meta-analysis, which have very low frequencies in the East African sample (less than 2%), are nominally significant and have strong effects on melanin levels. The lowest *p*-value correspond to the SNP rs35790407 (*p* = 3.87 × 10^− 3^, beta allele A = − 5.501). Unfortunately, some of the key variants in this region, including the non-synonymous SNP rs1042602 located in the *TYR* gene, are not present in the imputed East African dataset. Finally, two of the variants located in the *APBA2* region, rs148942115 and rs150641715, are nominally significant in the East African sample (rs150641715 *p* = 0.017, beta allele A = − 0.955).

**Table 4:** Replication of meta-analysis lead SNPs in an East African sample

SNP	EA	Chr	Position (GRCg37/hg19)	Genes^a^	*P*-value (meta-RE2 model)	*P*-value EAF	Beta EAF	SE EAF	MAF-EAF	Effect^b^ (meta-EAF)
rs35397	T	5	33951116	*SLC45A2*	1.98 × 10^− 24^	0.605	−0.403	0.780	0.068	--
rs10160510	T	11	88614324	*GRM5*	8.87 × 10^− 10^	0.347	− 1.681	1.784	0.012	--
rs3098576	C	15	27858408	*(GABRG3/OCA2)*	8.90 × 10^−09^	0.438	0.303	0.391	0.436	- +
rs1448484	A	15	28283441	*OCA2*	3.73 × 10^−10^	**4.43 × 10** ^**− 04**^	−1.467	0.417	0.363	--
rs1667392	C	15	28533565	*HERC2*	4.64 × 10^− 09^	NA	NA	NA	NA	
rs36194177	A	15	29118784	*(APBA2)*	4.95 × 10^−10^	NA	NA	NA	NA	
rs2636060	A	15	29425936	*FAM189A1*	5.71 × 10^− 10^	0.323	−0.393	0.397	0.390	--
rs1426654	A	15	48426484	*SLC24A5*	6.32 × 10^−39^	**6.89 × 10** ^**−61**^	−7.775	0.452	0.243	--
rs10416746	A	19	3563982	*(MFSD12)*	1.01 × 10^− 09^	**1.53 × 10** ^**− 04**^	2.019	0.532	0.167	+ +

*EA* Effect allele, *Chr* Chromosome, *RE2* Han and Eskin’s random effects model, *EAF* East Africa, *SE* Standard error, *MAF* Minor allele frequency. Significant *p*-values after Bonferroni correction are highlighted in bold

^a^For variants located in intergenic regions, nearby genes are indicated in parenthesis

^b^Direction of effect of the EA (− decreases melanin index, + increases melanin index)

### Follow-up of genome-wide signals described in recent studies

We also followed-up in our meta-analysis the genome-wide signals reported in the original GWAS in recently admixed populations (i.e. the samples that were included in this meta-analysis), as well as the recent GWAS in East and South African populations (Table 5) [10, 11]. Concordant effects were observed in all the previously described regions. For *SLC24A5, SLC45A2,* and *MFSD12,* at least some of the original signals described are also genome-wide significant in our meta-analysis. The lead signal reported in the *GRM5* gene [6] is also very close to genome-wide significance in our meta-analysis.

**Table 5:** Follow-up of previous GWAS genome-wide signals in our meta-analysis

SNP	EA	Chr	Position (GRCg37/hg19)	Gene	Reported *P*-value*	Ref.	*P*-value meta^†^	Beta meta	SE meta	Effect^g^ (study-meta)	*P*-value Cuba	*P*-value Cape Verde	*P*-value GALA II	*P*-value SAGE II
rs35395	T	5	33948589	*SLC45A2*	5.30 × 10^−08^	a	1.98 × 10^−24^	−0.321	0.032	--	1.26 × 10^− 12^	5.05 × 10^− 08^	1.28 × 10^− 05^	0.0094
rs16891982	G	5	33951693	*SLC45A2*	9.71 × 10^− 10^	b	2.13 × 10^−23^	−0.317	0.032	--	3.31 × 10^− 11^	3.04 × 10^− 07^	1.83 × 10^− 06^	0.0052
rs6602666	G	10	13606490	*BEND7-PRPF18*	4.58 × 10^− 09^	b	5.10 × 10^− 03^	0.129	0.046	+ +	0.9235	0.7941	2.53 × 10^− 05^	0.0139
rs11230664	C	11	61076372	*DDB1/ TMEM138*	2.10 × 10^− 09^	d	5.20 × 10^− 04^	0.121	0.035	+ +	0.3660	0.0004	0.7934	0.2027
rs7120594	T	11	61080557	*DDB1/ TMEM138*	1.20 × 10^− 08^	d	0.016	0.087	0.036	+ +	0.6213	0.0050	0.8055	0.4503
rs2513329	C	11	61106892	*DDB1*	5.20 × 10^−07^	c	2.20 × 10^− 04^	− 0.129	0.035	--	0.3281	0.0002	0.7934	0.1744
rs7948623	A	11	61137147	*DDB1/ TMEM138*	2.20 × 10^−11^	d	0.179	−0.074	0.055	--	NA	0.0922	0.8261	0.9972
rs10831496	G	11	88557991	*GRM5*	1.20 × 10^−08^	a	9.25 × 10^− 08^	0.168	0.032	+ +	0.0498	1.34 × 10^− 09^	0.0917	0.9202
rs1800404	T	15	28235773	*OCA2*	1.60 × 10^−08^	d	8.92 × 10^− 06^	−0.137	0.031	--	0.2368	0.0004	0.0618	0.0120
rs4932620	C	15	28514281	*HERC2*	3.20 × 10^−09^	d	5.03 × 10^− 03^	−0.258	0.092	--	NA	0.0014	0.7298	0.3685
rs4424881	T	15	29261716	*APBA2*	6.10 × 10^−09^	a	5.44 × 10^− 07^	0.158	0.032	+ +	0.8845	2.60 × 10^− 07^	0.0229	0.0149
rs1426654	G	15	48426484	*SLC24A5*	3.91 × 10^−24^; 2.62 × 10^−14^; 5.5 × 10^−62^; 9.8 × 10^−9^	a; b; d; e	6.32 × 10^− 39^	0.403	0.031	+ +	4.95 × 10^− 07^	2.87 × 10^−23^	6.39 × 10^− 08^	5.62 × 10^− 08^
rs139343937	A	16	80256441	*LOC102724084*	1.56 × 10^− 08^	f	3.45 × 10^− 06^	− 0.248	0.053	--	1.69 × 10^− 08^	0.3206	NA	NA
rs56203814	T	19	3544892	*MFSD12*	3.60 × 10^−18^	d	0.157	0.076	0.054	+ +	0.4032	0.1792	0.6323	0.1051
rs10424065	T	19	3545022	*MFSD12*	5.10 × 10^−20^	d	0.048	0.093	0.047	+ +	0.0184	0.0039	0.3854	0.0916
rs6510760	A	19	3565253	*MFSD12*	6.50 × 10^−15^	d	3.24 × 10^− 06^	0.164	0.035	+ +	0.8013	4.42 × 10^− 06^	0.2545	0.0096
rs112332856	C	19	3565599	*MFSD12*	3.80 × 10^−16^	d	2.31 × 10^− 08^	0.197	0.035	+ +	0.2740	1.21 × 10^− 06^	0.1604	0.0031

*EA* Effect allele, *Chr* Chromosome, *Ref* Reference of studied population

* *P*-value reported for rs2513329 corresponds to a Bayesian analysis

†All the *p*-values reported for the *BEND7-PRPF18, DDB1/TMEM138, OCA2/HERC2/APBA2, LOC102724084* and *MFSD12* regions are the *p*-values obtained after conditioning for the *SLC24A5* and *SLC45A2* signals

^a^Beleza et al., 2013 [6]

^b^Hernandez-Pacheco et al., 2017 [8]

^c^Lloyd-Jones et al., 2017 [7]

^d^Crawford et al.,2017 [10]

^e^Martin et al., 2017 [11]

^f^This study

^g^Direction of effect of the EA (− decreases melanin index, + increases melanin index)

In the *OCA2/HERC2/APBA2* region, the original signals reported in Cape Verde and East Africa do not reach genome-wide significance. As described above, we have observed other variants in this region that reach genome-wide significance in our meta-analysis. In the *DDB1/TMEM138* region, several of the lead SNPs reported in a recent Bayesian analysis of the Cape Verde sample [7], and a recent East African GWA study [10] are nominally significant in our meta-analysis. These results are primarily driven by the Cape Verde sample, and the *p*-values in the other samples are not significant. We could not confirm the genome-wide signals identified in the *BEND7/PRPF18* region in Puerto Rican and African-American samples [8] and the *LOC102724084* region in the Cuban sample. Finally, we also followed-up in our meta-analysis five suggestive variants reported in the recent GWA study in KhoeSan [11]: rs7866411 and rs2093835 in the *SMARCA2/VLDLR* region, rs34803545 and rs76413115 upstream of *TYRP1*, and rs210015 in *SNX13*. None of these SNPs was nominally significant in our meta-analysis.

### Follow-up of genome-wide signals associated with tanning response

We followed-up 20 genome-wide signals recently identified in a meta-analysis of tanning response including five large European samples [14] (Additional file 2: Table S6). Five variants located in known pigmentation genes are nominally significant in our meta-analysis: rs16891982 (*SLC45A2; p-*value = 2.13 × 10^− 23^), rs12203592 (*IRF4*; *p*-value = 1.4 × 10^− 3^), rs72917317 (*TPCN2*; *p*-value = 4.87 × 10^− 4^), rs1126809 (*TYR*; *p*-value = 1.73 × 10^− 3^), and rs12913832 (*HERC2*; *p*-value = 3.18 × 10^− 8^). One of the novel markers identified in the tanning response meta-analysis is also nominally significant in our conditional meta-analysis: rs35563099 (*EMX2*; *p*-value = 2.0 × 10^− 3^).

## Discussion

Here, we present the results of one of the largest association studies of skin pigmentation in recently admixed populations. Our meta-analysis includes over 2,000 unrelated admixed individuals from Cuba, Cape Verde, Puerto Rico and African Americans from the San Francisco Bay area. Although there are significant associations of African ancestry with skin pigmentation in all samples (Additional file 1: Figures S4-S7), there are considerable differences in the strength of the association, with *R*^2^ values ranging from 0.645 in the Cuban sample to 0.182 in the Puerto Rican sample. This is probably driven by differences in the population structure present in the samples. For the meta-analysis, we used summary data obtained after controlling for population structure (i.e. individual ancestry) and other relevant covariates in each sample.

The strongest signals observed in the meta-analysis correspond to SNPs in the well-known *SLC24A5* and *SLC45A2* genes. In both genes, the best candidates to explain the association are two non-synonymous variants: rs1426654 (*SLC24A5*) and rs16891982 *(SLC45A2).* The polymorphism rs16891982 is in very strong LD (*r*^2^ > 0.8) with another SNP in the *SLC45A2* gene, rs35397, which is the lead signal in our meta-analysis. The SNP rs16891982 is predicted to be damaging by both SIFT and Polyphen, so its effect may be mediated by structural changes in the protein coded by the *SLC45A2* gene, the membrane associated transporter protein (MATP), which works as a membrane transporter in melanosomes. Additionally, our eQTL analyses indicate that rs16891982 is associated with expression of the *SLC45A2* gene (*p* = 4.78 × 10^− 7^), although we cannot exclude the possibility that this association is driven by potential LD with other variants not genotyped in this region. The derived G allele, which is associated with lower melanin levels, is associated with an increased expression of the *SLC45A2* gene. *SLC24A5* rs1426654 and *SLC45A2* rs16891982 are the variants with the strongest effect on skin pigmentation described in human populations and have been reported in numerous association studies [4–11, 15–17]. Further, *SLC45A2* rs16891982 has also been associated with tanning response and skin cancer in several studies [15, 18–20].

Genome-wide association signals were also identified in a region encompassing the genes *GRM5* and *TYR* on chromosome 11. Another clear candidate to explain this association is the non-synonymous rs1042602 located in the *TYR* gene (*p* = 9.2 × 10^− 10^), which is in strong LD (*r*^2^ > 0.70) with our lead SNP (rs10160510, *p* = 3.36 × 10^− 10^) and it is predicted to be damaging by both SIFT and Polyphen. Enhancer histone marks, H3K4me1 and H3K27ac, have been identified in foreskin melanocyte primary cells overlapping two variants that are in high LD with rs1042602: rs12285584 and rs12295166. Two GWAS have previously reported associations of rs1042602 with pigmentary traits, such as skin pigmentation [21] and freckles [22]. Importantly, there seem to be more than one independent signal in the *GRM5/TYR* region. When carrying out association tests in the Cape Verde sample conditioning for rs1042602, or rs10160510, there are several genome-wide significant markers in the *GRM5* gene that remain strongly associated with skin pigmentation (e.g. rs492312, *p*-value conditioning for rs1042602 = 3.82 × 10^− 6^, *p*-value conditioning for rs10160510 = 1.65 × 10^− 4^). The SNP rs492312 is in strong LD (*r*^2^ > 0.90) with markers that overlap with enhancer histone marks and/or DNase hypersensitive sites in foreskin fibroblast primary skin cells [23]. We also found support for the role of variants within the *GRM5* gene in the follow-up of our genome-wide significant signals in the East African sample (Additional file 2: Table S6). Many of these variants, although found at very low frequencies in the East African sample (less than 2%), are nominally associated with skin pigmentation, and the estimated effects on skin pigmentation are very large (e.g. rs35790407, MAF = 1.1%, *p* = 3.87 × 10^− 3^, beta = − 5.501; rs11021131, MAF = 1.6%, *p* = 0.012, beta = − 3.957). We hypothesize that the association of variants located on the *GRM5* gene with pigmentation may be due to their regulation of expression of the *TYR* gene, which is a critical gene in melanogenesis.

We also observed strong associations of variants located on chromosome 19, near the *MFSD12* gene, with skin pigmentation. The role of this gene on skin pigmentation has only been recently described. Crawford et al. [10] reported variants associated with skin pigmentation in two different regions on chromosome 19, one within and another upstream of the *MFSD12* locus, in an East African sample. Additionally, they showed, using experiments in mouse melanocyte cultures and zebrafish and mice animal models, that *MFSD12* has a functional role on pigmentation. Of note, one of the SNPs identified in our meta-analysis, rs112332856 (2.31 × 10^− 8^), is one of the genome-wide variants originally reported by [10]. This SNP is associated with expression of the *MFSD12* gene. In particular, the rs112332856 C allele, which is associated with darker skin pigmentation, is associated with decreased expression of *MFSD12*, and this is consistent with experiments in mouse melanocyte culture that indicate that depletion of *MFSD12* increases eumelanin biogenesis in melanocytes.

Additionally, we observed multiple genome-wide signals in a broad region of chromosome 15 overlapping the genes *OCA2/HERC2/APBA2*. A cluster of variants is located upstream of the *OCA2* gene (lead SNP rs3098576, *p* = 8.9 × 10^− 9^) and the markers in this region are not in strong LD with other genome-wide signals identified in the *OCA2/HERC2/APBA2* region. The posterior probabilities of having an effect (M-values) are high in all the samples of the meta-analysis, and there is no evidence of heterogeneity in effect sizes. However, we could not replicate these results in the East African sample, in spite of the fact that the relevant variants are present at high frequencies in the sample. It will be necessary to carry out additional studies to elucidate the potential role of this region on skin pigmentation variation.

Aside from the aforementioned region, we identified genome-wide clusters in a segment of approximately 1 Megabase that includes markers located within the *OCA2* gene, the *HERC2* gene and within or nearby the *APBA2* and *FAM189A1* genes. These clusters are located more than 250 Kb apart, but the markers in different clusters are in LD (D’ > 0.8; Additional file 1: Figures S10-S17). Identifying the putative causal variants in this region is challenging for two reasons: the aforementioned LD between markers, and problems with imputation of variants between the *HERC2* and *APBA2* region, which is probably due to the presence of segmental duplications in this genomic region [24]. For example, some of the genome-wide signals identified in our meta-analysis were not present in the imputed data used to evaluate gene expression in melanocytes, or in the imputed East African dataset.

In spite of these challenges, there are several functional candidates in the *OCA2/HERC2/APBA2* region. Within *OCA2*, our lead SNP is rs1448484. This variant has been identified in studies of signatures of selection as one of the markers with the highest continental differentiation in human populations [25, 26]. The derived G allele, which is strongly associated with light skin pigmentation, is almost fixed in non-African populations, whereas the ancestral A allele is the most common allele in African populations. This polymorphism has been associated with skin pigmentation in a previous study [27]. Additionally, this marker and linked *OCA2* polymorphisms overlap with enhancer histone marks and DNase hypersensitive sites in foreskin fibroblast primary skin cells [23]. In the recent GWA study in East Africa, the main signal in the *OCA2* region was the synonymous SNP rs1800404. However, this SNP is not in very strong LD with rs1448484 (*r*^2^ < 0.60), and is not genome-wide significant in our meta-analysis (rs1800404 *p* = 8.92 × 10^− 6^ vs. rs1448484 *p* = 3.73 × 10^− 10^). The high M-values observed in the meta-analysis indicate that the probabilities of having an effect are high for rs1448484 in the four samples analyzed. It would be important to carry out additional studies in substantially larger admixed and African samples in order to explore the reasons for the different association patterns identified in the *OCA2* region.

Within *HERC2*, our lead signal rs1667392 (*p* = 4.64 × 10^− 9^) is in strong LD with rs12913832 (*p* = 3.18 × 10^− 8^), an intronic SNP that has a known regulatory effect on *OCA2* expression by disrupting the interaction between an enhancer located on *HERC2* and the *OCA2* promoter [28, 29]. This SNP is strongly associated with blue eye color [28–33]. Our melanocyte eQTL data fully supports the functional effects described for rs12913832; the derived G allele, which is associated with light pigmentation in our meta-analysis, shows a strong association with reduced *OCA2* expression (*p* = 3.14 × 10^− 23^). Of note, in addition to the extensively reported association with blue eye color, rs12913832 has been associated with skin pigmentation and hair color in previous studies [15, 22, 34–37].

We also observed genome-wide significant signals downstream of *HERC2*, near the *APBA2* gene. This region was previously reported in the original GWAS in the Cape Verde sample [6], although the top signals reported in that study are not the same as those identified in our meta-analysis. Interestingly, the lead SNP in the meta-analysis (rs36194177, *p* = 4.95 × 10^− 10^), and variants in LD with this SNP (rs142415892 and rs148942115), overlap with enhancer histone marks and DNase hypersensitive sites in several tissues, and there is an enrichment for enhancers in foreskin keratinocyte primary cells in skin [23]. Unfortunately, none of our genome-wide signals in the *APBA2* region was present in the imputed data used to evaluate gene expression in melanocyte cultures, so we could not evaluate potential associations of these variants with gene expression of relevant genes, and more particularly, *OCA2*. However, three of the variants were present in the East African imputed dataset (rs142415892, rs148942115, and rs150641715), and two of them are nominally associated with skin pigmentation (e.g. rs150641715, *p* = 0.017).

The final genome-wide signals identified in this region are located within the *FAM189A1* gene (lead SNP rs2636060, *p* = 5.7 × 10^− 10^). To our knowledge, this region has not been described in previous pigmentation association studies. The posterior probabilities of having an effect (M-values) are high in all the samples of the meta-analysis, and there is no evidence of heterogeneity in effect sizes, as observed for the region located upstream of *OCA2*. However, the two genome-wide signals are not significant in the East African sample, and there is no overlap of this region with putative regulatory regions active in skin cells [23].

Given the pattern of LD observed in the *OCA2/HERC2/APBA2* region, we carried out additional conditional and haplotype tests to evaluate if the signals observed in the meta-analysis may be due to a single causal SNP, or to independent effects. These tests point to the presence of independent variants. When conditioning for each of the lead SNPs from the four relevant clusters, rs1448484 (*OCA2*), rs1667392 (*HERC2*), rs36194177 (near *APBA2*) and rs2636060 (*FAM189A1*), the *p*-values of the other markers remain nominally significant. The haplotype tests also support at least partially independent effects. As expected, the haplotype associated with the strongest reduction in melanin levels is the haplotype harboring the four variants common in European populations (ACGA). However, there are other haplotypes that do not include some of the variants common in Europe that are also associated with lower melanin levels, and the haplotype patterns are quite similar in Cape Verde and Cuba. Controlling for the haplotype ACGA in the Cape Verdean sample, there is still a marginal association with skin pigmentation, which is driven by the haplotypes GGGA and AGGA.

Given that some variants that have an effect on constitutive pigmentation have been found to be associated with facultative pigmentation (i.e. tanning ability) [14, 18], we followed-up in our meta-analysis the genome-wide significant markers identified in a recent tanning response GWAS [14]. These results are depicted in Additional file 2: Table S7. One of the novel markers identified in the aforementioned GWAS, located nearby *EMX2* on chromosome 10 is nominally significant in our meta-analysis of constitutive pigmentation (rs35563099, *p*-value = 0.002). *EMX2* is known to be involved in the synthesis of melanin, and it has been shown that overexpression of the Emx2 homologous gene causes loss of pigmentation by downregulating the Mitf gene in mice [38]. Our study indicates that this gene is involved both in tanning response and constitutive skin pigmentation.

When our manuscript was under review, an article was published describing the results of a GWAS of skin and iris pigmentation in a sample of more than 6,000 Latin Americans [9]. Some of the findings of this study are very similar to those described in the present study. More particularly, the authors also reported multiple independent signals within the *GRM5/TYR* and *OCA2/HERC2* regions. In the *GRM5/TYR* region, Adhikari et al. [9] reported three signals: rs7118677 (*GRM5*), rs1042602 (*TYR*) and rs1126809 (*TYR*). In the *OCA2/HERC2* region, they reported five signals: rs4778219 (*OCA2*), rs1800407 (*OCA2*), rs1800404 (*OCA2*), rs12913832 (*HERC2*) and rs4778249 (*HERC2*). We followed up all the genome-wide signals reported by Adhikari et al. [9] in our meta-analysis (Additional file 2: Table S8). Several of these signals are also the lead signals identified in our study (e.g. rs1042602 in *TYR*, rs12913832 at *HERC2*), clearly pointing to the same causal variants. However, other lead variants are different to our lead SNPs in the relevant regions (Table 2; Additional file 2: Table S8), and Adhikari et al. [9] do not report genome-wide significant variants within the *APBA2* region. Some of these discrepancies may be driven by the demographic differences between both datasets. The GWAS on Latin Americans [9] includes samples from Chile, Mexico, and Peru, which have primarily Native American and European ancestry, with very small African ancestral contributions (< 5%), and samples from Brazil and Colombia, which have primarily European ancestry, with substantially lower Native American (12.1 and 29.2%, respectively) and African contributions (~ 10% in both samples). In contrast, the samples included in our study are characterized by a wide distribution of West African and European ancestry, without (e.g. Cape Verde) or with very small Native American ancestry (Cuba, Puerto Rico, African Americans).

Interestingly, Adhikari et al. [9] also reported a non-synonymous genome-wide signal within the gene *MFSD12*: rs2240751. However, this missense variant is common only in East Asian and Native American populations, and is different from the variants reported in the recent GWAS in East African populations [10] and those we found in the present study, which reflects the different demographic composition of the samples. Clearly, there have been independent mutational events within this gene in African and East Asian/Native American groups.

In summary, in this meta-analysis we have identified multiple regions significantly associated with skin pigmentation variation in admixed populations. All of these regions have been reported before. However, our analyses indicate that in some of these regions there are multiple independent signals. We have not identified any novel pigmentation regions in our study. This may be related to limited power to identify variants with moderate effects on skin pigmentation. Further meta-analytic efforts including larger sample sizes will be needed to uncover this type of variants, and to elucidate the reasons driving the heterogeneity in effect sizes observed for some of the signals identified in this study.

## Conclusions

We identified five genome-wide significant regions in a meta-analysis of skin pigmentation in recently admixed populations with primarily European and African ancestry, and explored if the markers observed in these regions are associated with the expression of relevant pigmentary genes in human melanocyte cultures. In three of the regions identified in the meta-analysis (*SLC24A5*, *SLC45A2*, and *GRM5/TYR*), the association seems to be driven by non-synonymous variants (rs1426654, rs16891982, and rs1042602, respectively). The rs16891982 polymorphism is strongly associated with the expression of the *SLC45A2* gene. In the *GRM5/TYR* region, in addition to the rs1042602 non-synonymous SNP located on the *TYR* gene, variants located in the nearby *GRM5* gene have an independent effect on pigmentation, possibly through regulation of gene expression of the *TYR* gene. We were also able to confirm the effect of a recently described variant near the *MFSD12* gene (rs112332856) on skin pigmentation. This variant has been shown to alter the expression of the nearby gene [10]. Finally, our analyses support the presence of several variants with effects on skin pigmentation in the *OCA2/HERC2/APBA2* region on chromosome 15. A clear causal candidate is rs12913832 within *HERC2*, which has a profound influence on *OCA2* expression in eye, hair, and skin pigmentation. However, our results also point out the presence of other independent variants modulating melanin levels in the genes *OCA2* and *APBA2*.

Overall, our meta-analysis and other GWAS in multiple population groups provide fascinating insights on the genetic architecture and evolutionary history of pigmentary traits. 1) There is evidence of independent functional mutations within the same genes in different populations (i.e. convergent evolution). Different variants within *OCA2/HERC2* are associated with light pigmentation in European and East Asian populations [39–42]. Independent mutations within the *MFSD12* gene are associated with skin pigmentation variation in populations with diverse African ancestry and Native American/East Asian ancestry (e.g. this study, [9, 10]). 2) There is evidence of multiple independent signals in the same genomic regions, such as *GRM5/TYR* and *OCA2/HERC2,* within the same population group (e.g. this study, [9]). 3) There is evidence of pleiotropic effects for many significantly associated variants. For example, the *HERC2* rs12913832 SNP, which was originally associated with eye color, also plays a role in skin and hair color pigmentation [15, 22, 34–37]. Additionally, many of the variants associated with skin pigmentation are also associated with tanning response (e.g. *EMX2*) (this study, [9, 14]). Unfortunately, studies focusing on tanning response are still in their infancy. 4) Finally, there is evidence pointing to the mechanisms of action by which genetic polymorphisms exert a role on pigmentation variation (e.g. effects on protein structure, regulation of gene expression).

As new studies emerge with increasing sample sizes and more comprehensive population coverages, it will be possible to gain an even deeper understanding of the main events that shaped the diversity of pigmentary traits and allowed our species to adapt to different latitudinal environments.

## Methods

### Cuban dataset

The Cuban sample comprises 1,019 individuals representing all Cuban provinces [12]. Skin pigmentation was measured using the DSM II Derma Spectrometer (Cortex Technology, Hadsund, Denmark) in triplicate for each participant, from the inner skin of their upper right arm. The DNA samples were genotyped using the Infinium PsychChip v1.0 and v1.1 arrays (Illumina Inc., San Diego, California, U.S.A.), at the Statens Serum Institut in Copenhagen, Denmark. We performed quality control (QC) steps to remove samples and markers, according to the following criteria, Sample QC: 1/removal of samples with missing call rates < 0.95, 2/removal of samples that were outliers in Principal Component Analysis (PCA) plots, 3/removal of duplicates or samples with sex discrepancies, 4/removal of samples that were outliers for heterozygosity, and 5/removal of related individuals (pi-hat > 0.2). Marker QC: 1/removal of markers with genotype call rate < 0.95, 2/removal of markers with Hardy-Weinberg *p*-values < 10^− 6^, 3/removal of Insertion/Deletion (Indel) markers, 4/removal of markers with allele frequencies < 0.01, 5/removal of markers not present in the 1000 Genomes reference panel, or that do not match on chromosome, position and alleles, 6/removal of A/T or G/C SNPs with MAF > 40% in the 1000 Genomes American (AMR) samples, and 7/removal of SNPs with allele frequency differences > 20% between the study sample and the 1000 Genomes AMR reference sample. The final call set includes a total of 292,549 autosomal SNPs across 762 unrelated Cuban individuals with skin pigmentation data. Haplotype phasing was done with the program SHAPEIT2 [43]. Imputation of non-genotyped SNPs was done at the Sanger Imputation Service, using the Positional Burrows-Wheeler Transform algorithm [44], and the samples of the 1000 Genomes Project Phase 3 (1KGP) as reference haplotypes.

### Datasets of other admixed samples

We carried out a meta-analysis based on the GWAS results for the Cuban sample, as well as imputed genome-wide data available for three additional admixed samples. The first sample comprises 684 individuals from the archipelago of Cape Verde [6]. The second sample includes 373 African Americans from the San Francisco Bay Area, who were recruited as part of the Study of African Americans, Asthma, Genes and Environments (SAGE II) [8]. The third sample comprises 285 unrelated individuals from Puerto Rico, who participated in the study of Genes-environment and Admixture in Latin Americans (GALA II) [8]. Further details for each dataset, such as information about skin pigmentation measurements, microarray genotyping and imputation procedure, are provided in Additional file 4: Text S1.

### Statistical analyses

#### Inverse normal transformation

In order to combine the GWAS results of the four samples in a meta-analysis, quantitative pigmentation measures (M-index values) were transformed prior to statistical analysis using the rank-based inverse normal transformation (Blom method). In a first step, a linear regression was carried out using pigmentation values as the dependent variable, and sex, age, and relevant PCA scores as independent variables. The residuals of this regression were then transformed using the inverse normal transformation, which was used as outcome variable for the association tests.

#### Association tests

For the Cuban and Cape Verde samples association tests were performed in SNPTEST v2 [45], using expected genotype counts (e.g. genotype dosages) and an additive model. For the SAGE II and GALA II samples, association tests were performed with a linear Wald test (q.lm) implemented in the software EPACTS 3.2.6 [46] using genotype dosages and an additive model. Two sets of association analysis were done in each sample: standard association tests, and also association tests conditioning for the variants rs1426654 (*SLC24A5*) and rs35397 (*SLC45A2*), which are known to have very strong effects on pigmentation and had very low *p*-values in the four samples analyzed.

#### Meta-analysis of association results

The summary statistics from the four GWAS were used to run a meta-analysis using the program METASOFT [47, 48]*.* This program implements a fixed effects model based on inverse-variance-weighted effect size and also Han and Eskin’s random effects model (RE2), which has been shown to have higher statistical power to detect associations under heterogeneity than the conventional random effects model based on inverse-variance-weighted effect size [47]. Additionally, METASOFT provides estimates of the posterior probability that an effect exists in each study (M-values) [47]. Small M-values indicate that the study is predicted to not have an effect, large M-values indicate that the study is predicted to have an effect, and intermediate M-values indicate ambiguous results.

#### Linkage disequilibrium, conditional and haplotype analyses

To gain a better understanding of the multiple clusters identified in the *OCA2/HERC2/APBA2* region on chromosome 15, we used the software Haploview [49], to explore the LD patterns (r^2^ and D’ values) between each pair of genome-wide signals found within this region in the Cuban and Cape Verde samples, as well as the Puerto Rican (PUR) and African American in Southwest US (ASW) samples included in the 1KGP.

To test whether the lead signals in each cluster are independent from each other, we carried out conditional analyses for this region in the Cuban and Cape Verde samples. Specifically, we conducted several association tests conditioning for each of the lead signals identified in each cluster. The genetic markers used were rs1448484 in *OCA2*, rs1667392 in *HERC2*, rs36194177 in *APBA2,* and rs2636060 in *FAM189A1*.

Additionally, haplotype association analyses were conducted in the Cuban and Cape Verde samples using the program PLINK v1.07 [50], which are the samples for which we had individual genome-wide data. Specifically, we employed omnibus tests to evaluate if there are significant effects of the haplotypes on the phenotype. These tests also provide estimates of effect sizes with respect to a reference haplotype. We defined the haplotype GGAG as the reference, given that it contains the alleles that are most frequent in African populations, which are all associated with higher melanin index values in the meta-analysis. Finally, we carried out haplotype tests controlling for specific haplotypes using PLINK –chap –control option. Again, these analyses were done based on haplotypes including rs1448484 in *OCA2*, rs1667392 in *HERC2*, rs36194177 in *APBA2,* and rs2636060 in *FAM189A1*.

### Testing association with gene expression based on RNA-seq data

To test for associations between gene expression and genetic variation, we carried out an eQTL analysis based on transcriptome and genotype data from primary cultures of human melanocytes, isolated from foreskin of 106 healthy newborn males [13]. DNA samples were genotyped on Illumina OmniExpress arrays and genotypes were subsequently imputed using the Michigan Imputation Server [51], based on the 1KGP Phase 3 reference panel and using SHAPEIT for phasing [43]. Post-imputation variants with MAF < 0.01 or imputation quality scores (*R*^2^) < 0.3 were removed from the final analysis. PCA was performed to capture population structure using the struct.pca module of Genotyping Library and Utilities [52]. Analysis with the program ADMIXTURE indicated that 84 individuals are of predominant European ancestry, 3 individuals of predominant African ancestry, 3 individuals of predominant East Asian ancestry, 12 individuals show mixed European and African ancestry, and 4 individuals have mixed European and Asian ancestry [13]. RNA sequencing was performed on a HiSeq2500 using version 4 chemistry to achieve a minimum of 45 million 126 base paired reads. The updated pipeline originally published by University of North Carolina for processing TCGA RNA-sequencing data was used to analyze the RNA- sequence data generated from the samples. STAR [53] was used for aligning reads. RSEM [54] was used to quantify the gene expression to transcripts per million (TPM) and then quantile normalization was applied to the TPM in all samples to get the final RSEM value. To account for hidden factors driving expression variabilities, a set of covariates were further identified using the PEER method [55] and applied to calculate the normalized expression matrices. Associations between variant genotypes and gene expression levels were evaluated using linear regression implemented in FastQTL [56], using the set of covariates identified with the PEER method and the PC scores as covariates. Genetic variants located within +/− 1 Mb of the transcription start sites for each gene were tested for cis-eQTL effects of the corresponding gene. More detailed information about the gene expression analysis is available in [13].

### Annotation of genome-wide association signals identified in the meta-analysis

The genome-wide association signals identified in the meta-analysis were annotated using the SNPnexus website [57]. This site provides numerous annotations, including potential effects on protein function (e.g. SIFT and PolyPhen), conservation scores (e.g. phastCons and GERP), and a range of scores for non-coding variants (e.g. CADD, fitCons, EIGEN, FATHMM, GWAVA, DeepSEA, FunSeq2, and ReMM). We also explored potential regulatory effects in HaploReg v4 [58] and RegulomeDB [59].

## Additional files

Additional file 1: **Figure S1. **Distribution of M-index values in the Cuban sample. **Figure S2.** Manhattan plot depicting the results of the GWAS for the Cuban sample. **Figure S3. **QQ plot from the initial GWAS for the Cuban sample. **Figures S4-S7.** Correlations of African individual proportions and melanin index in the different samples. **Figures S8-S9.** QQ plots from the initial and conditional meta-analyses. **Figures S10-S17.** LD (r2 and D’) among the genome-wide significant SNPs in the *OCA2/HERC2/APBA2* region of chromosome 15 in the Cuba and Cape Verde samples, and in the ASW and PUR populations of the 1KGP. **Figures S18-S27.** Regional plots of the *OCA2/HERC2/APBA2* top markers on the region of chromosome 15. (DOCX 11908 kb)

Additional file 2: **Table S1.** Top genome-wide significant SNPs in meta-analysis and on each independent study. **Table S2.** Top genome-wide significant SNPs in conditional meta-analysis and on each independent study. **Table S3.** Association tests conditioning for each of the lead signals in the *OCA/HERC2/APBA2* region of Chromosome 15, for the Cape Verde and Cuba samples, separately. **Tables S4-S5.** Omnibus tests in the Cape Verde and Cuba samples separately, based on haplotypes from our lead signals (rs1448484, rs1667392, rs36194177, rs2636060). **Table S6.** Follow-up results of all genome-wide signals identified from our meta-analysis in an East African sample. **Table S7.** Follow-up of tanning response signals in our meta-analysis. **Table S8.** Follow-up of skin pigmentation signals identified in a recent GWAS in a large Latin American sample in our meta-analysis. (DOCX 75 kb)

Additional file 3: The first sheet (‘Cuba’) includes the results obtained from the GWAS conducted in the Cuban sample, the second sheet (‘Meta’) includes the initial meta-analysis results, and the third sheet (‘Meta-cond’) includes the conditional meta-analysis results. (XLSX 843 kb)

Additional file 4: **Text S1** Supplementary information on the methods followed for the datasets included in the meta-analysis (Cape Verde, SAGE II, GALA II). (DOCX 28 kb)

## Data Availability

We provide the list of all genome-wide (*p* < 5 × 10^− 8^) and suggestive (*p* < 10^− 5^) signals identified in the meta-analysis as a supplementary information file (Additional file 3). The complete summary data of the meta-analysis is available from the corresponding author upon request.

## Abbreviations

1KGP: 1000 genomes project
eQTL: Expression quantitative trait loci
GALA II: Genes-environment and Admixture in Latin Americans
GWAS: Genome-wide association studies
LD: Linkage disequilibrium
MAF: Minor allele frequency
SAGE II: Study of African Americans, Asthma, Genes and Environment
SNP: Single nucleotide polymorphism
