# Supplementary material for: Meta-analysis of GWA studies provides new insights on the genetic architecture of skin pigmentation in recently admixed populations
Source: BMC Genet. 2019 Jul 17;20:59. doi: 10.1186/s12863-019-0765-5 (PMC6637524; doi:10.1186/s12863-019-0765-5)
Supplement: Supplementary file 1 — Figure S1. Distribution of M-index values in the Cuban sample. Figure S2. Manhattan plot depicting the results of the GWAS for the Cuban sample. Figure S3. QQ plot from the initial GWAS for the Cuban sample. Figures S4-S7. Correlations of African individual proportions and melanin index in the different samples. Figures S8-S9. QQ plots from the initial and conditional meta-analyses. Figures S10-S17. LD (r2 and D’) among the genome-wide significant SNPs in the OCA2/HERC2/APBA2 region of chromosome 15 in the Cuba and Cape Verde samples, and in the ASW and PUR populations of the 1KGP. Figures S18-S27. Regional plots of the OCA2/HERC2/APBA2 top markers on the region of chromosome 15. (DOCX 11908 kb) [file 12863_2019_765_MOESM1_ESM.docx]

**Supplementary Figures**


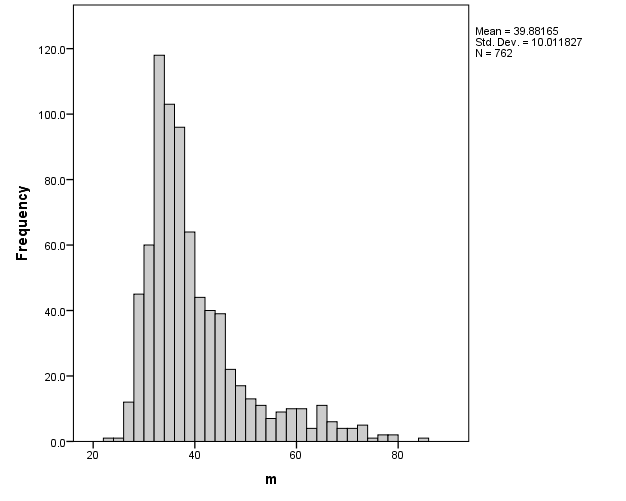


**Figure S1.** Distribution of M-index values (m) in the Cuban sample.


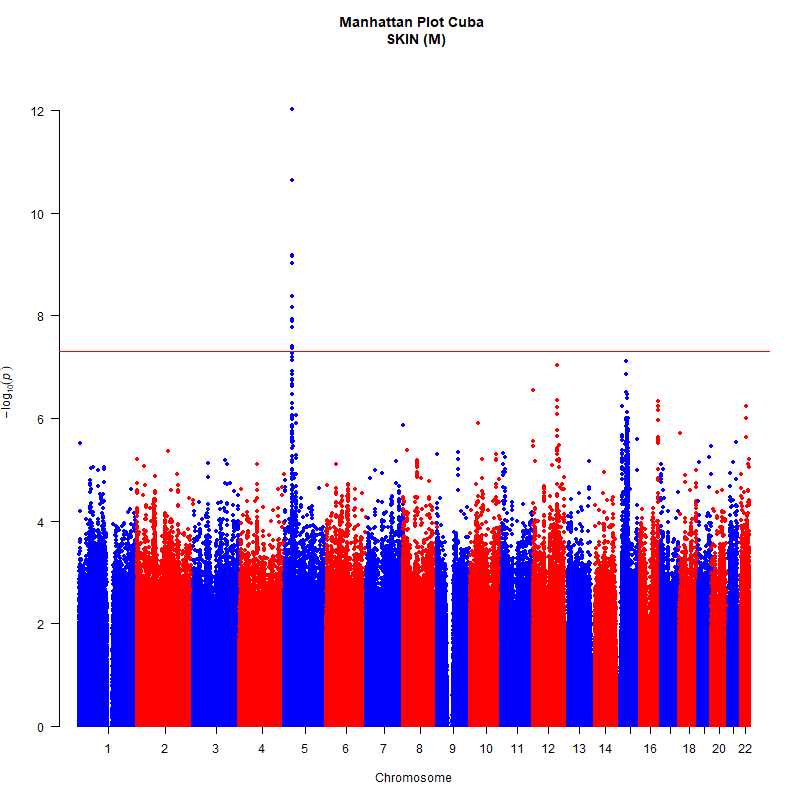


**Figure S2.** Manhattan plot depicting the results of the GWAS for the Cuban sample. The red horizontal is the genome-wide significance threshold (p = 5 x 10^-8^).


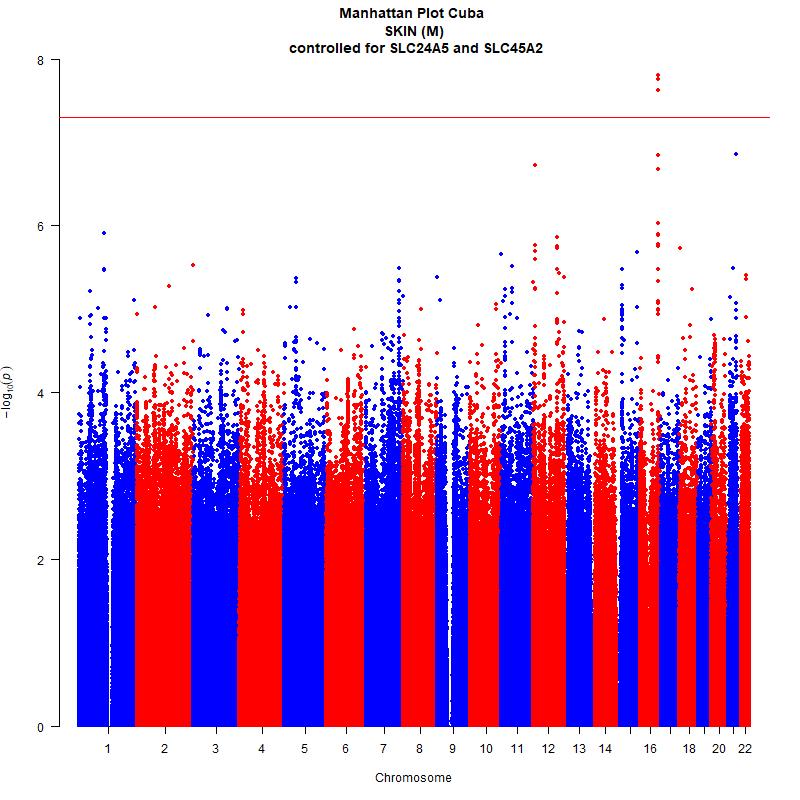


Figure S3. Manhattan plot depicting the results of the conditional GWAS for the Cuban sample.


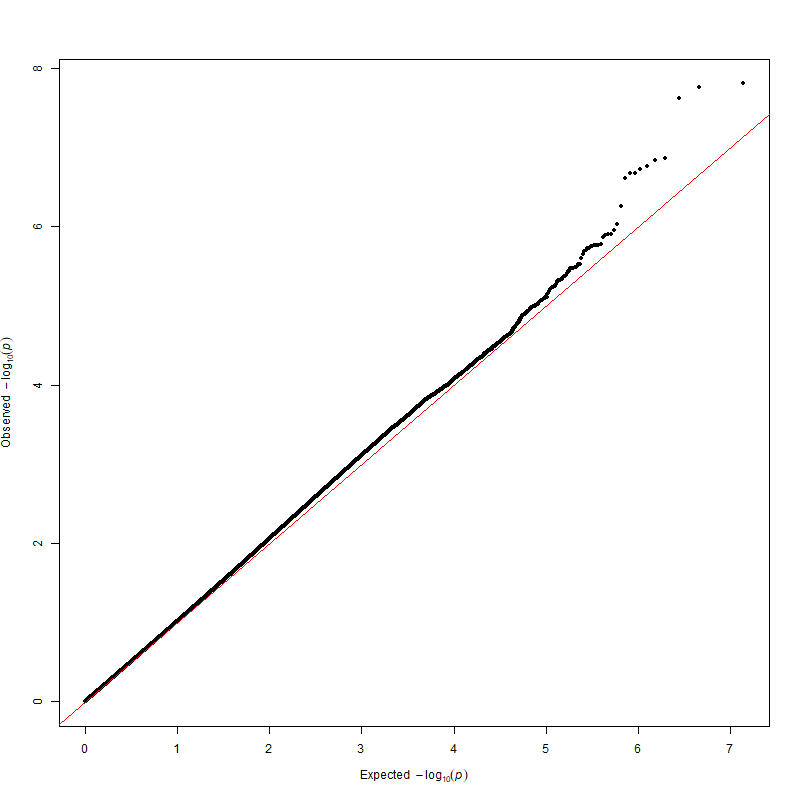


Figure S5. QQ plot of conditional GWAS results for the

Cuban sample. λGC = 1.027.


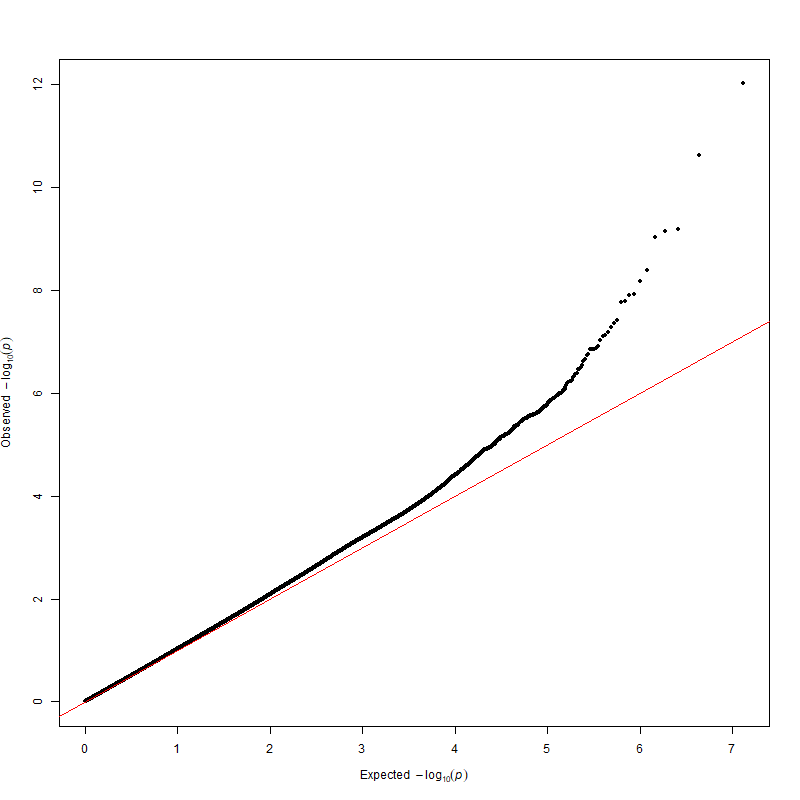


**Figure S3.** QQ plot of -log_10_(p) values from the initial GWAS for the Cuban sample. λGC = 1.046.


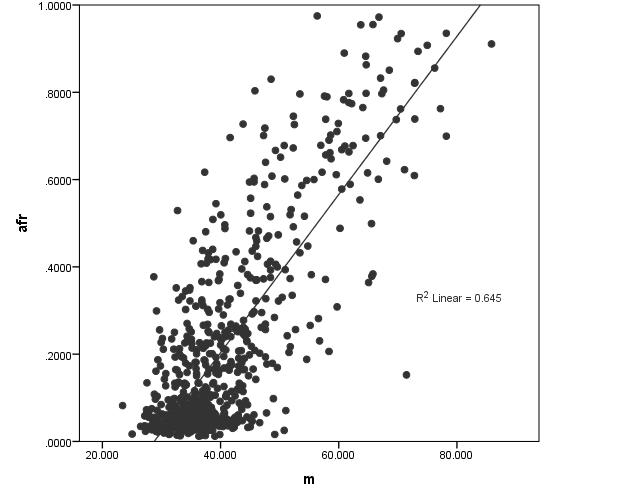


**Figure S4.** Correlation of African individual proportions (afr) and melanin index (m) in the Cuban sample.


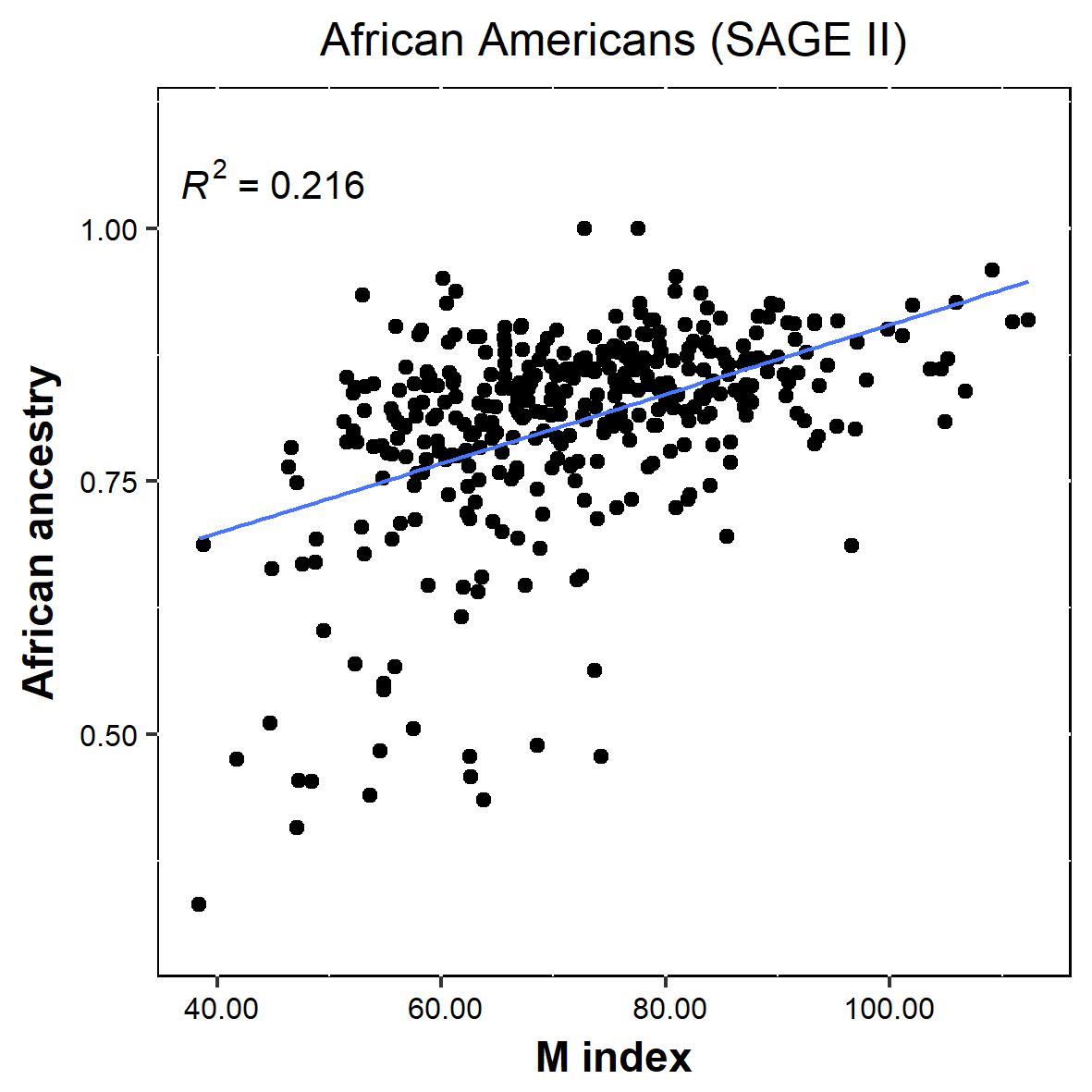


**Figure S5.** Correlation of African individual proportions and melanin index in the African American sample (SAGE II).


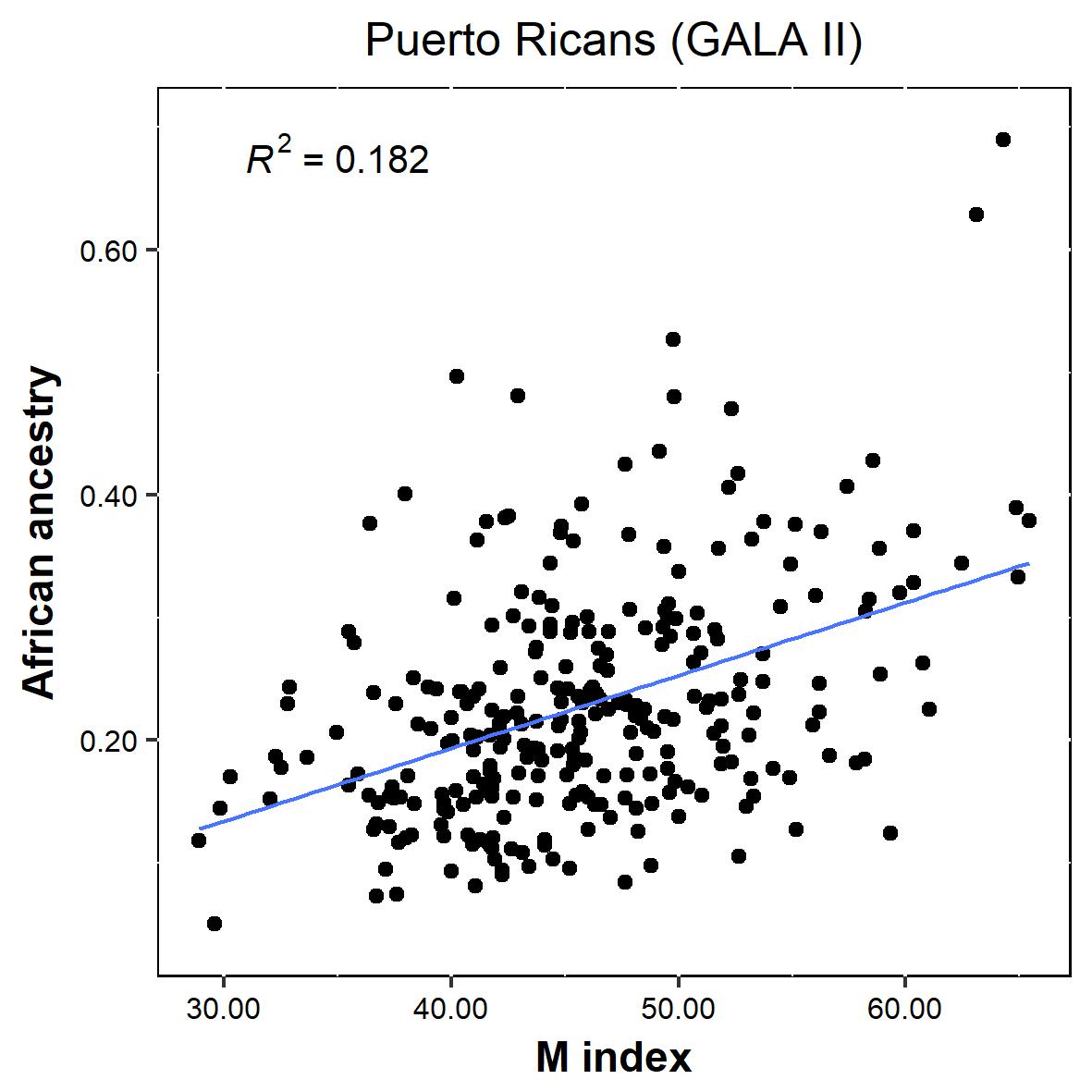


**Figure S6.** Correlation of African individual proportions and melanin index in the Puerto Rican sample (GALA II).


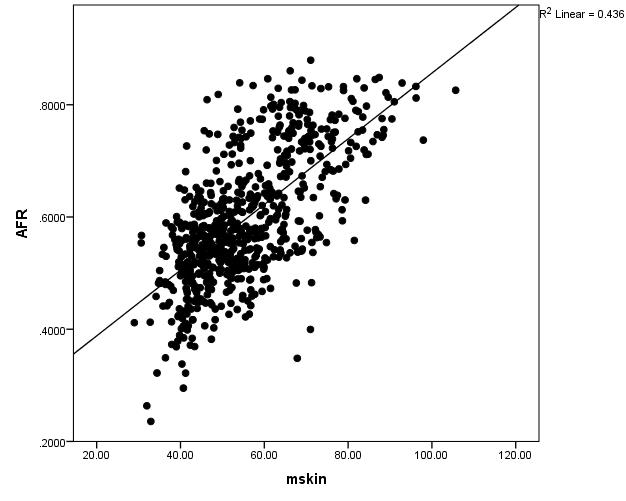


**Figure S7.** Correlation of African individual proportions (afr) and melanin index (m) in the Cape Verde sample.


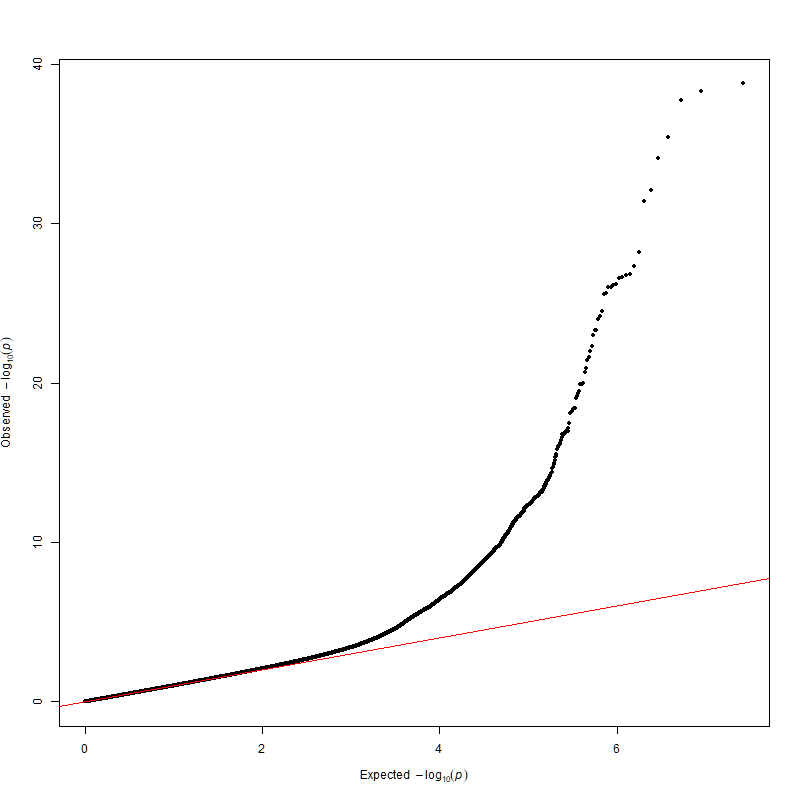


**Figure S8.** QQ plot of -log_10_(p) values from the meta-analysis. λGC = 1.05.


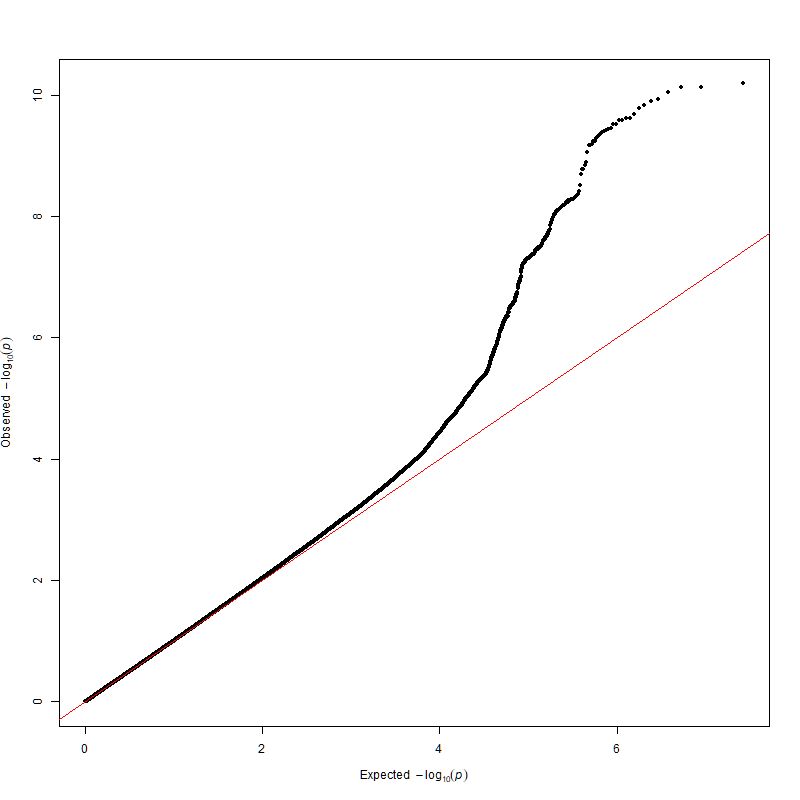


**Figure S9.** QQ plot of -log_10_(p) values from the conditional meta-analysis. λGC = 1.016.


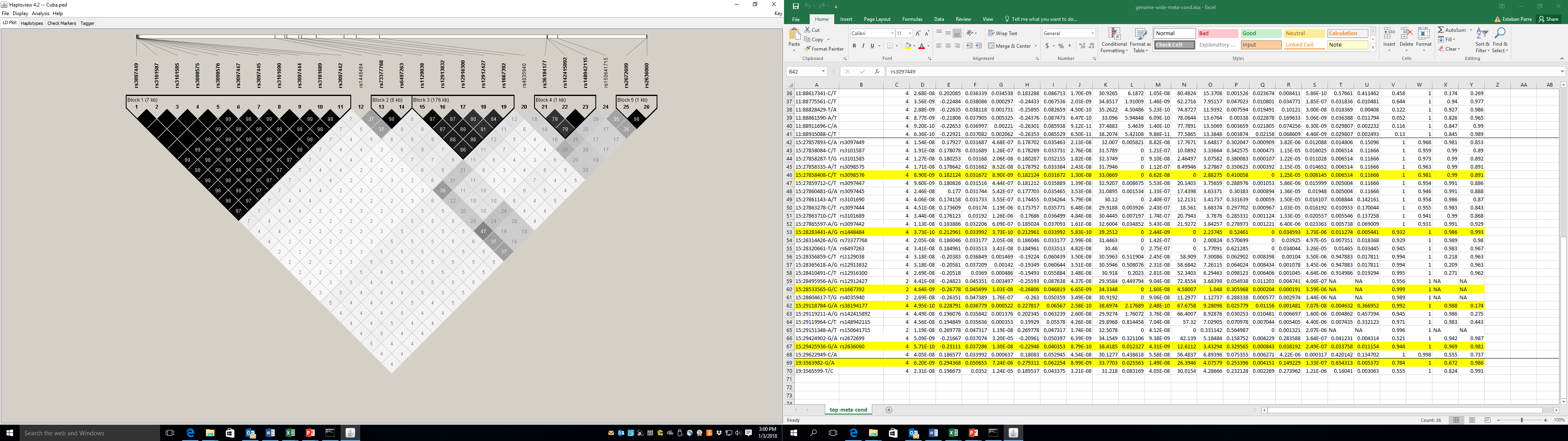


**Figure S10.** LD (r^2^) among the genome-wide significant SNPs in the *OCA2/HERC2/APBA2* region of chromosome 15 for the Cuban sample. Percentage of LD depicted for each pair of markers. Dark colors represent high LD, whereas lighter colors represent low LD.


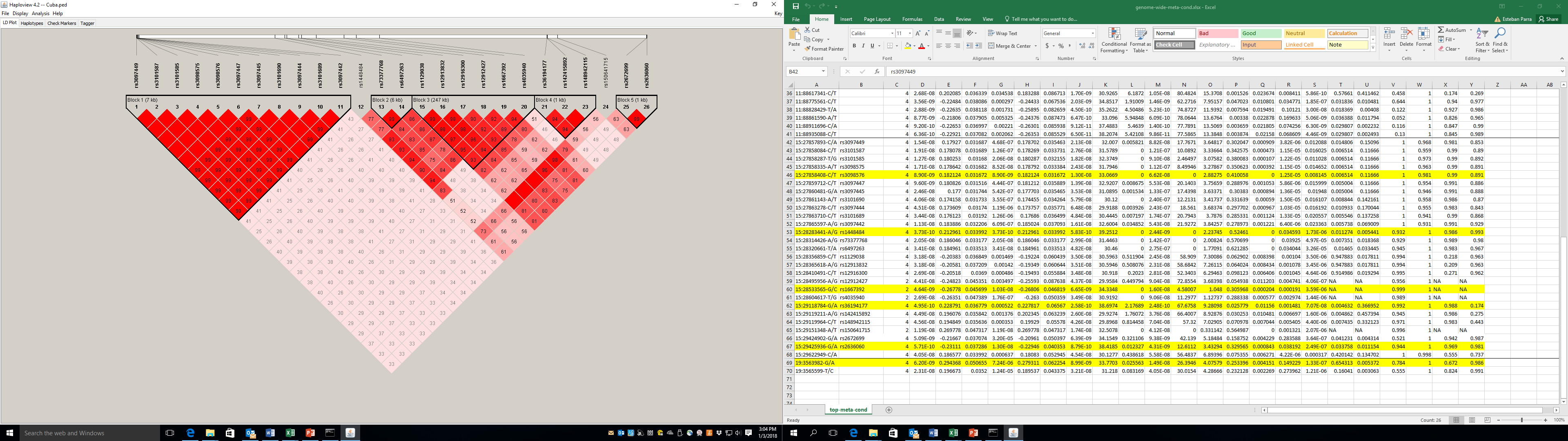


**Figure S11.** LD (D’) among the genome-wide significant SNPs in the *OCA2/HERC2/APBA2* region of chromosome 15 for the Cuban sample. Percentage of LD depicted for each pair of markers. Dark colors represent high LD, whereas lighter colors represent low LD.


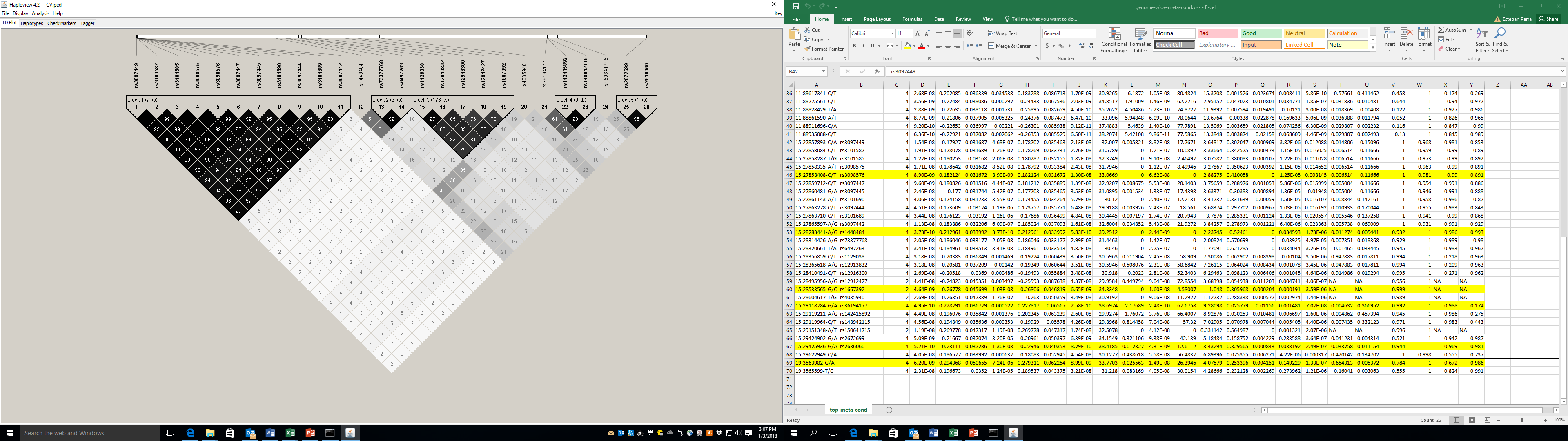


**Figure S12.** LD (r^2^) among the genome-wide significant SNPs in the *OCA2/HERC2/APBA2* region of chromosome 15 for the Cape Verde sample. Percentage of LD depicted for each pair of markers. Dark colors represent high LD, whereas lighter colors represent low LD.


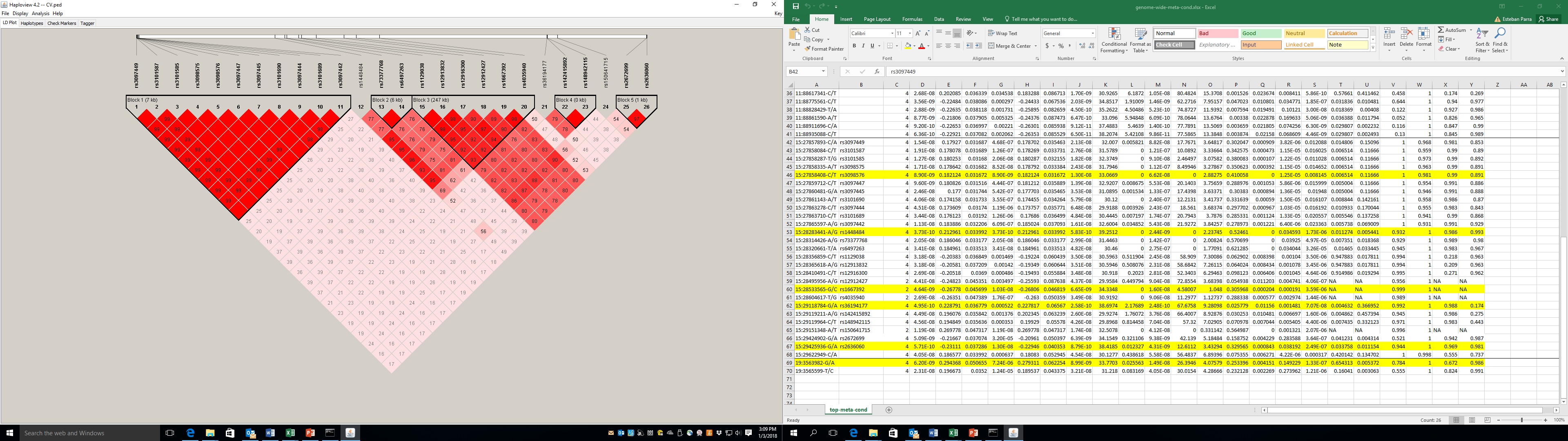


**Figure S13.** LD (D’) among the genome-wide significant SNPs in the *OCA2/HERC2/APBA2* region of chromosome 15 for the Cape Verde sample. Percentage of LD depicted for each pair of markers. Dark red colors represent high LD, whereas lighter red colors represent low LD.


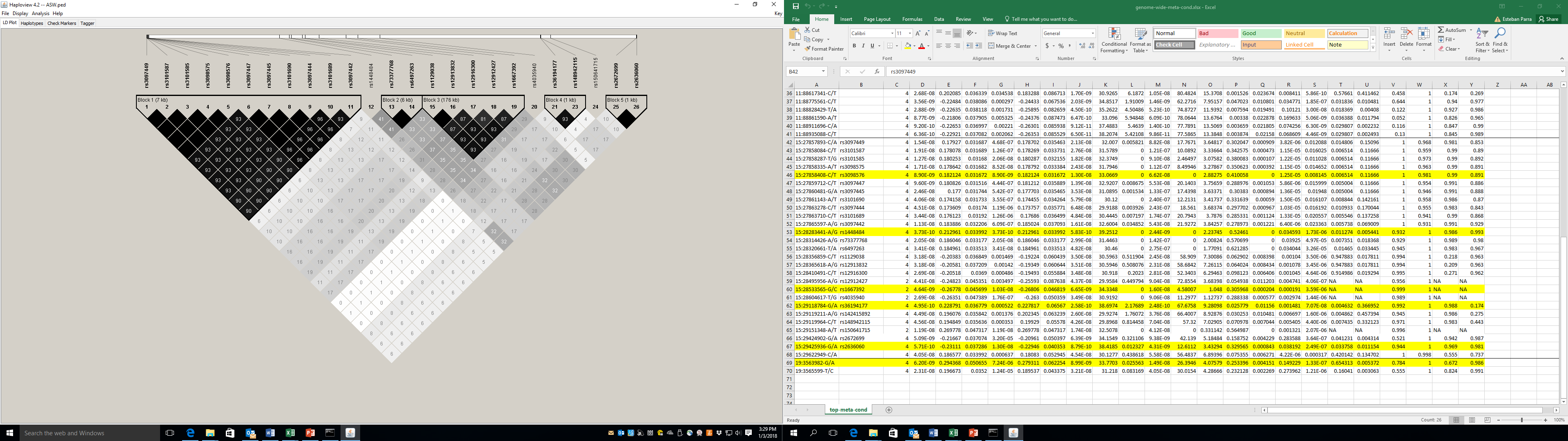


**Figure S14.** LD (r^2^) among the genome-wide significant SNPs in the *OCA2/HERC2/APBA2* region of chromosome 15, for the Americans of African Ancestry in SW USA (ASW) population from the 1KGP. Percentage of LD depicted for each pair of markers. Dark colors represent high LD, whereas lighter colors represent low LD.


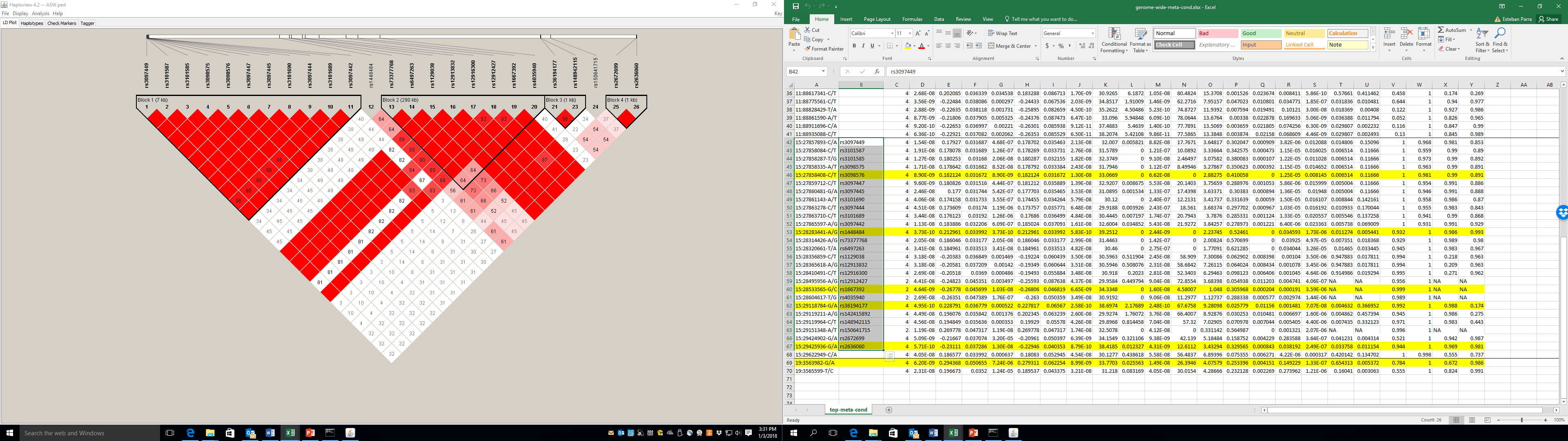


**Figure S15.** LD (D’) among the genome-wide significant SNPs in the *OCA2/HERC2/APBA2* region of chromosome 15, for the Americans of African Ancestry in SW USA (ASW) population from the 1KGP. Percentage of LD depicted for each pair of markers. Dark red colors represent high LD, whereas lighter red colors represent low LD.


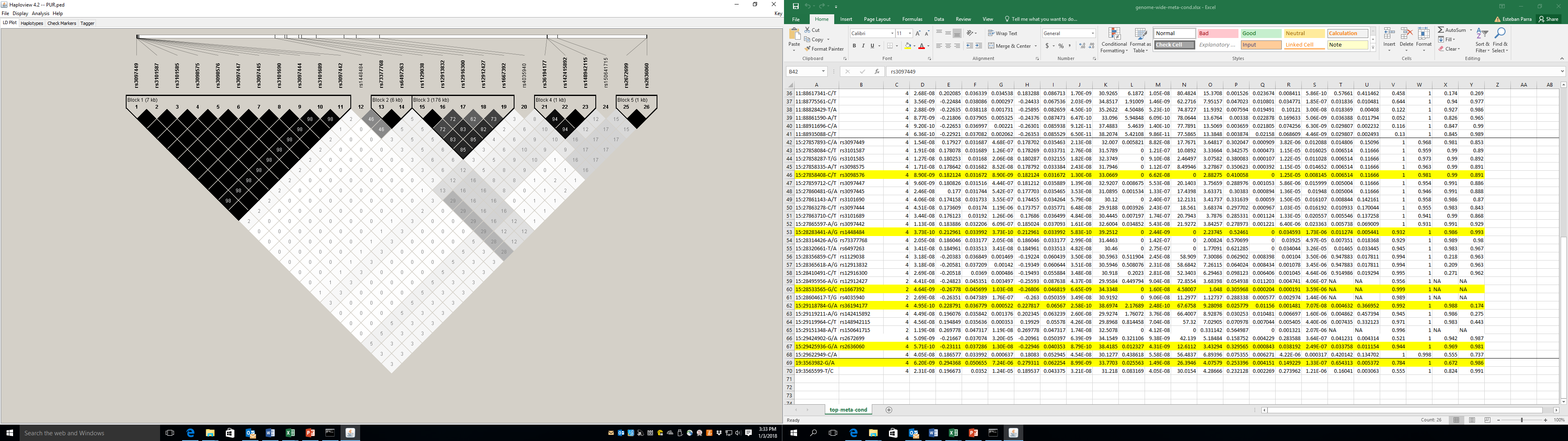


**Figure S16.** LD (r^2^) among the genome-wide significant SNPs in the *OCA2/HERC2/APBA2* region of chromosome 15, for the Puerto Ricans from Puerto Rico (PUR) population from the 1KGP. Percentage of LD depicted for each pair of markers. Dark colors represent high LD, whereas lighter colors represent low LD.


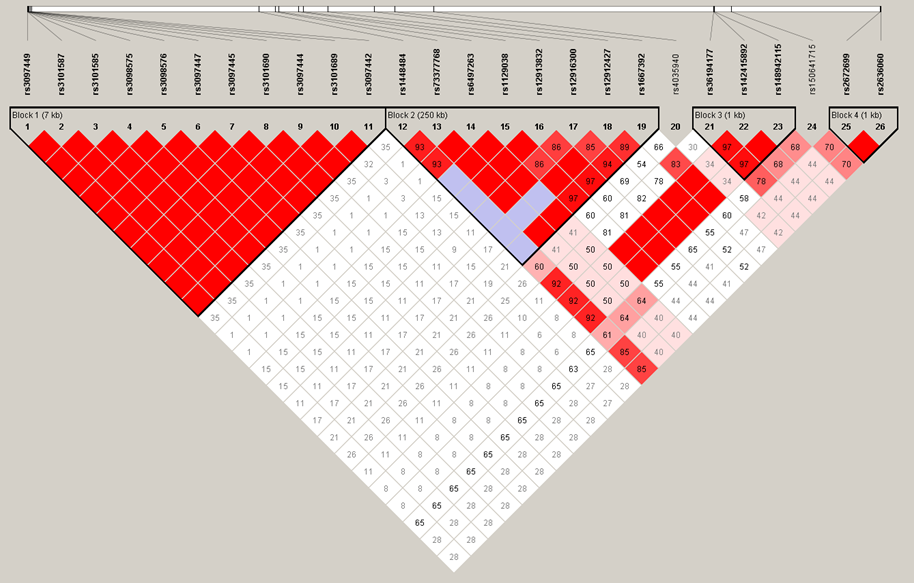


**Figure S17.** LD (D’) among the genome-wide significant SNPs in the *OCA2/HERC2/APBA2* region of chromosome 15, for the Puerto Ricans from Puerto Rico (PUR) population from the 1KGP. Percentage of LD depicted for each pair of markers. Dark red colors represent high LD, whereas lighter red colors represent low LD.

**Figure S18.** Regional plot of the *OCA2/HERC2/APBA2* region of chromosome 15, showing the LD patterns (measured as r^2^) between the marker rs3098576 upstream of the *OCA2* gene and other markers in the region in African populations.

**Figure S19.** Regional plot of the *OCA2/HERC2/APBA2* region of chromosome 15, showing the LD patterns (measured as r^2^) between the marker rs3098576 upstream of the *OCA2* gene and other markers in the region in European populations.

**Figure S20.** Regional plot of the *OCA2/HERC2/APBA2* region of chromosome 15, showing the LD patterns (measured as r^2^) between the marker rs1448484 within the *OCA2* gene and other markers in the region in African populations.

**Figure S21.** Regional plot of the *OCA2/HERC2/APBA2* region of chromosome 15, showing the LD patterns (measured as r^2^) between the marker rs1448484 within the *OCA2* gene and other markers in the region in European populations.

**Figure S22.** Regional plot of the *OCA2/HERC2/APBA2* region of chromosome 15, showing the LD patterns (measured as r^2^) between the marker rs12913832 within the *HERC2* gene and other markers in the region in African populations. No data were available for the lead SNP within the *HERC2* region (rs1667392) in the Locuszoom website.

**Figure S23.** Regional plot of the *OCA2/HERC2/APBA2* region of chromosome 15, showing the LD patterns (measured as r^2^) between the marker rs12913832 within the *HERC2* gene and other markers in the region in European populations. No data were available for the lead SNP within the *HERC2* region (rs1667392) in the Locuszoom website.

**Figure S24.** Regional plot of the *OCA2/HERC2/APBA2* region of chromosome 15, showing the LD patterns (measured as r^2^) between the marker rs36194177 upstream of the *APBA2* gene and other markers in the region in African populations.

**Figure S25.** Regional plot of the *OCA2/HERC2/APBA2* region of chromosome 15, showing the LD patterns (measured as r^2^) between the marker rs36194177 upstream of the *APBA2* gene and other markers in the region in European populations.

**Figure S26.** Regional plot of the *OCA2/HERC2/APBA2* region of chromosome 15, showing the LD patterns (measured as r^2^) between the marker rs2636060 within the *FAM189A1* gene and other markers in the region in African populations.

**Figure S27.** Regional plot of the *OCA2/HERC2/APBA2* region of chromosome 15, showing the LD patterns (measured as r^2^) between the marker rs2636060 within the *FAM189A1* gene and other markers in the region in European populations.
