# Supplementary material for: Meta-analysis of GWA studies provides new insights on the genetic architecture of skin pigmentation in recently admixed populations
Source: BMC Genet. 2019 Jul 17;20:59. doi: 10.1186/s12863-019-0765-5 (PMC6637524; doi:10.1186/s12863-019-0765-5)
Supplement: Supplementary file 2 — Table S1. Top genome-wide significant SNPs in meta-analysis and on each independent study. Table S2. Top genome-wide significant SNPs in conditional meta-analysis and on each independent study. Table S3. Association tests conditioning for each of the lead signals in the OCA/HERC2/APBA2 region of Chromosome 15, for the Cape Verde and Cuba samples, separately. Tables S4-S5. Omnibus tests in the Cape Verde and Cuba samples separately, based on haplotypes from our lead signals (rs1448484, rs1667392, rs36194177, rs2636060). Table S6. Follow-up results of all genome-wide signals identified from our meta-analysis in an East African sample. Table S7. Follow-up of tanning response signals in our meta-analysis. Table S8. Follow-up of skin pigmentation signals identified in a recent GWAS in a large Latin American sample in our meta-analysis. (DOCX 75 kb) [file 12863_2019_765_MOESM2_ESM.docx]

**Supplementary Tables**

**Table S1. Top genome-wide significant SNPs in meta-analysis and on each independent study.** ^a^: Fixed effects model, as computed in Metasoft. ^b^: Han and Eskin’s Random Effects Model. Chr: chromosome; EA: effect allele; NEA: non-effect allele; SD: Standard Deviation; M: posterior probability of an existent effect on each study; I^2^: heterogeneity statistic.

| **SNP** | **Chr** | **Position (GRCg37/hg19)** | **EA/ NEA** | **Fixed Effects Model^a^** | | | **Random Effects Model^b^** | **Cochran’s Q** | | **I^2^** |
| --- | --- | --- | --- | --- | --- | --- | --- | --- | --- | --- |
|  |  |  |  | **P-value** | **Beta** | **SD** | **P-value** | **Q** | **P-value** |  |
| rs35397 | 5 | 33951116 | T/G | 1.98E-24 | -0.321 | 0.032 | 4.76E-24 | 1.837 | 0.607 | 0 |
| rs10160510 | 11 | 88614324 | T/A | 7.11E-08 | -0.215 | 0.040 | 5.49E-08 | 6.841 | 0.077 | 56.145 |
| rs3098576 | 15 | 27858408 | T/C | 1.90E-08 | 0.179 | 0.032 | 2.74E-08 | 3.219 | 0.359 | 6.795 |
| rs1448484 | 15 | 28283441 | G/A | 5.97E-09 | 0.199 | 0.034 | 8.77E-09 | 0.431 | 0.934 | 0 |
| rs1667392 | 15 | 28533565 | C/G | 7.78E-09 | -0.222 | 0.038 | 8.83E-09 | 6.728 | 0.081 | 55.413 |
| rs36194177 | 15 | 29118784 | A/G | 6.86E-09 | 0.214 | 0.037 | 4.68E-09 | 8.344 | 0.039 | 64.045 |
| rs2636060 | 15 | 29425936 | A/G | 6.97E-11 | -0.244 | 0.037 | 1.13E-10 | 2.234 | 0.525 | 0 |
| rs1426654 | 15 | 48426484 | G/A | 6.32E-39 | 0.403 | 0.031 | 1.63E-39 | 12.449 | 0.006 | 75.901 |
| rs10416746 | 19 | 3563982 | A/G | 4.43E-09 | 0.298 | 0.051 | 6.56E-09 | 3.435 | 0.329 | 12.669 |

continued:

| **SNP** | **Cuba** | | **Cape Verde** | | **GALA II** | | **SAGE II** | | **Effect Allele Frequency in each population** | | | | | |
| --- | --- | --- | --- | --- | --- | --- | --- | --- | --- | --- | --- | --- | --- | --- |
|  | **P-value** | **M** | **P-value** | **M** | **P-value** | **M** | **P-value** | **M** | **Europe** | **Cuba** | **Cape Verde** | **GALA II** | **SAGE II** | **Africa** |
| rs35397 | 1.26E-12 | 1 | 5.05E-08 | 1 | 1.28E-05 | 1 | 9.37E-03 | 0.982 | 0.92 | 0.549 | 0.323 | 0.438 | 0.217 | 0.082 |
| rs10160510 | 1.65E-02 | 0.875 | 6.68E-07 | 1 | 1.76E-01 | 0.813 | 5.80E-02 | 0.929 | 0.306 | 0.283 | 0.168 | 0.209 | 0.069 | 0.011 |
| rs3098576 | 9.55E-06 | 1 | 1.15E-02 | 0.972 | 1.03E-02 | 0.986 | 1.34E-01 | 0.874 | 0.171 | 0.328 | 0.456 | 0.350 | 0.528 | 0.612 |
| rs1448484 | 1.21E-02 | 0.985 | 4.39E-05 | 1 | 4.91E-02 | 0.961 | 6.45E-03 | 0.992 | 0.008 | 0.165 | 0.417 | 0.120 | 0.642 | 0.803 |
| rs1667392 | 1.42E-03 | 0.993 | 9.46E-07 | 1 | 6.77E-01 | 0.359 | 1.73E-02 | 0.967 | 0.753 | 0.306 | 0.212 | NA | NA | 0.139 |
| rs36194177 | 1.39E-02 | 0.92 | 8.24E-07 | 1 | 1.17E-03 | 0.996 | 3.10E-01 | 0.328 | 0.073 | 0.208 | 0.421 | 0.240 | 0.484 | 0.658 |
| rs2636060 | 3.19E-03 | 0.996 | 7.20E-07 | 1 | 1.79E-02 | 0.983 | 1.51E-02 | 0.971 | 0.966 | 0.786 | 0.544 | 0.842 | 0.502 | 0.238 |
| rs1426654 | 4.95E-07 | 1 | 2.87E-23 | 1 | 6.39E-08 | 1 | 5.62E-08 | 1 | 0.003 | 0.288 | 0.506 | 0.421 | 0.762 | 0.923 |
| rs10416746 | 1.65E-01 | 0.761 | 6.89E-07 | 1 | 6.44E-01 | 0.669 | 8.76E-04 | 0.997 | 0 | 0.081 | 0.248 | 0.039 | 0.183 | 0.238 |

**Table S2. Top genome-wide significant SNPs in conditional meta-analysis and on each independent study.**

| **SNP** | **Chr** | **Position (GRCg37/hg19)** | **EA/ NEA** | **Genes^a^** | **Fixed Effects Model^b^** | | | **Random Effects Model^c^** | **Cochran’s Q** | | **I^2^** |
| --- | --- | --- | --- | --- | --- | --- | --- | --- | --- | --- | --- |
|  |  |  |  |  | **P-value** | **Beta** | **SD** | **P-value** | **Q** | **P-value** |  |
| rs10160510 | 11 | 88614324 | T/A | *GRM5* | 3.36E-10 | -0.248 | 0.039 | 7.54E-11 | 11.078 | 0.011 | 72.920 |
| rs3098576 | 15 | 27858408 | T/C | *(GABRG3/OCA2)* | 8.90E-09 | 0.182 | 0.032 | 1.30E-08 | 2.883 | 0.410 | 0.000 |
| rs1448484 | 15 | 28283441 | G/A | *OCA2* | 3.73E-10 | 0.213 | 0.034 | 5.83E-10 | 2.237 | 0.525 | 0.000 |
| rs1667392 | 15 | 28533565 | C/G | *HERC2* | 4.64E-09 | -0.268 | 0.046 | 6.65E-09 | 1.048 | 0.306 | 4.580 |
| rs36194177 | 15 | 29118784 | A/G | *(APBA2)* | 4.95E-10 | 0.229 | 0.037 | 2.58E-10 | 9.281 | 0.026 | 67.676 |
| rs2636060 | 15 | 29425936 | A/G | *FAM189A1* | 5.71E-10 | -0.231 | 0.037 | 8.79E-10 | 3.433 | 0.330 | 12.611 |
| rs10416746 | 19 | 3563982 | A/G | *(MFSD12)* | 1.01E-09 | 0.309 | 0.051 | 1.55E-09 | 3.947 | 0.267 | 24.002 |

continued:

| **SNP** | **Cuba** | | | **Cape Verde** | | | **GALA II** | | | | **SAGE II** | | | |
| --- | --- | --- | --- | --- | --- | --- | --- | --- | --- | --- | --- | --- | --- | --- |
|  | **P-value** | **Beta** | **M** | **P-value** | **Beta** | **M** | **P-value** | **Beta** | **M** | **P-value** | | **Beta** | **M** |  |
| rs10160510 | 9.50E-03 | -0.15 | 0.783 | 1.36E-09 | -0.44 | 1 | 1.12E-01 | -0.16 | 0.791 | 3.27E-02 | | -0.33 | 0.947 |  |
| rs3098576 | 1.25E-05 | 0.24 | 1 | 8.15E-03 | 0.14 | 0.981 | 6.51E-03 | 0.23 | 0.99 | 1.17E-01 | | 0.12 | 0.891 |  |
| rs1448484 | 3.46E-02 | 0.14 | 0.932 | 1.73E-06 | 0.25 | 1 | 1.13E-02 | 0.28 | 0.986 | 5.44E-03 | | 0.21 | 0.993 |  |
| rs1667392 | 1.91E-04 | -0.23 | 0.999 | 3.59E-06 | -0.32 | 1 | 0.777 | -0.03 | 0.235 | 0.0063 | | -0.27 | 0.985 |  |
| rs36194177 | 1.48E-03 | 0.22 | 0.992 | 7.07E-08 | 0.36 | 1 | 4.63E-03 | 0.27 | 0.988 | 3.67E-01 | | 0.06 | 0.174 |  |
| rs2636060 | 3.82E-02 | -0.16 | 0.944 | 2.49E-07 | -0.32 | 1 | 3.38E-02 | -0.24 | 0.969 | 1.12E-02 | | -0.18 | 0.981 |  |
| rs10416746 | 1.49E-01 | 0.17 | 0.758 | 1.33E-07 | 0.39 | 1 | 6.44E-01 | 0.09 | 0.656 | 8.47E-04 | | 0.27 | 0.997 |  |

continued:

| **SNP** | **Effect Allele Frequency in each population** | | | | | | |
| --- | --- | --- | --- | --- | --- | --- | --- |
|  | **Europe** | **Cuba** | **Cape Verde** | **GALA II** | **SAGE II** | **Africa** |  |
| rs10160510 | 0.306 | 0.283 | 0.168 | 0.209 | 0.069 | 0.011 |  |
| rs3098576 | 0.171 | 0.328 | 0.456 | 0.350 | 0.528 | 0.612 |  |
| rs1448484 | 0.008 | 0.165 | 0.417 | 0.120 | 0.642 | 0.803 |  |
| rs1667392 | 0.753 | 0.306 | 0.212 | NA | NA | 0.139 |  |
| rs36194177 | 0.073 | 0.208 | 0.421 | 0.240 | 0.484 | 0.658 |  |
| rs2636060 | 0.966 | 0.786 | 0.544 | 0.842 | 0.502 | 0.238 |  |
| rs10416746 | 0 | 0.081 | 0.248 | 0.039 | 0.183 | 0.238 |  |

The table presents the results of the meta-analysis after conditioning for *SLC24A5* rs1426654 and *SLC45A2* rs35397, which had very strong effects in the original meta-analysis (p = 6.32 x 10^-39^, and rs35397, p = 1.98 x 10^-24^, respectively). ^a^: For variants located in intergenic regions, nearby genes are indicated in parenthesis. ^b^: Fixed effects model, as computed in Metasoft. ^c^: Han and Eskin’s Random Effects Model. Chr: chromosome; EA: effect allele; NEA: non-effect allele; SD: Standard Deviation; M: posterior probability of an existent effect on each study; I^2^: heterogeneity statistic; NA: marker not genotyped.

**Table S3. Association tests conditioning for each of the lead signals in the *OCA2/HERC2/APBA2* region of Chromosome**

**15, for the Cape Verde and Cuba samples, separately.** Chr: chromosome; NEA: non-effect allele; EA: effect allele.

| **Cape Verde** | **Chr** | **Position (GRCg37/hg19)** | **NEA** | **EA** | **EAF** | **Original P-value** | **Original Beta** | **P-value cond** | **Beta cond** |
| --- | --- | --- | --- | --- | --- | --- | --- | --- | --- |
| **conditioning for rs1448484** |  | | | | | | | | |
| rs1448484 | 15 | 28283441 | A | G | 0.416 | 4.39E-05 | 0.215 | NA | NA |
| rs1667392 | 15 | 28533565 | G | C | 0.212 | 9.46E-07 | -0.341 | 6.90E-04 | -0.243 |
| rs36194177 | 15 | 29118784 | G | A | 0.420 | 8.24E-07 | 0.332 | 5.41E-03 | 0.168 |
| rs2636060 | 15 | 29425936 | G | A | 0.543 | 7.20E-07 | -0.306 | 6.32E-04 | -0.203 |
| **conditioning for rs1667392** |  | | | | | | | | |
| rs1448484 | 15 | 28283441 | A | G | 0.416 | 4.39E-05 | 0.215 | 1.91E-02 | 0.132 |
| rs1667392 | 15 | 28533565 | G | C | 0.212 | 9.46E-07 | -0.341 | NA | NA |
| rs36194177 | 15 | 29118784 | G | A | 0.420 | 8.24E-07 | 0.332 | 2.21E-03 | 0.167 |
| rs2636060 | 15 | 29425936 | G | A | 0.543 | 7.20E-07 | -0.306 | 5.21E-04 | -0.191 |
| **conditioning for rs36194177** |  | | | | | | | | |
| rs1448484 | 15 | 28283441 | A | G | 0.416 | 4.39E-05 | 0.215 | 3.96E-02 | 0.124 |
| rs1667392 | 15 | 28533565 | G | C | 0.212 | 9.46E-07 | -0.341 | 5.64E-04 | -0.238 |
| rs36194177 | 15 | 29118784 | G | A | 0.420 | 8.24E-07 | 0.332 | NA | NA |
| rs2636060 | 15 | 29425936 | G | A | 0.543 | 7.20E-07 | -0.306 | 7.66E-04 | -0.192 |
| **conditioning for rs2636060** |  | | | | | | | | |
| rs1448484 | 15 | 28283441 | A | G | 0.416 | 4.39E-05 | 0.215 | 6.72E-02 | 0.108 |
| rs1667392 | 15 | 28533565 | G | C | 0.212 | 9.46E-07 | -0.341 | 1.81E-03 | -0.218 |
| rs36194177 | 15 | 29118784 | G | A | 0.420 | 8.24E-07 | 0.332 | 1.08E-02 | 0.146 |
| rs2636060 | 15 | 29425936 | G | A | 0.543 | 7.20E-07 | -0.306 | NA | NA |

Continued:

| **Cuba** | **Chr** | **Position** | **NEA** | **EA** | **EAF** | **Original P-value** | **Original Beta** | **P-value cond** | **Beta cond** |
| --- | --- | --- | --- | --- | --- | --- | --- | --- | --- |
| **conditioning for rs1448484** |  | | | | | | | | |
| rs1448484 | 15 | 28283441 | A | G | 0.165 | 1.21E-02 | 0.163 | NA | NA |
| rs1667392 | 15 | 28533565 | G | C | 0.305 | 1.42E-03 | -0.196 | 2.00E-02 | -0.134 |
| rs36194177 | 15 | 29118784 | G | A | 0.208 | 1.39E-02 | 0.171 | 3.03E-01 | 0.071 |
| rs2636060 | 15 | 29425936 | G | A | 0.785 | 3.19E-03 | -0.226 | 6.66E-01 | -0.036 |
| **conditioning for rs1667392** |  | | | | | | | | |
| rs1448484 | 15 | 28283441 | A | G | 0.165 | 1.21E-02 | 0.163 | 7.22E-02 | 0.118 |
| rs1667392 | 15 | 28533565 | G | C | 0.305 | 1.42E-03 | -0.196 | NA | NA |
| rs36194177 | 15 | 29118784 | G | A | 0.208 | 1.39E-02 | 0.171 | 1.54E-01 | 0.089 |
| rs2636060 | 15 | 29425936 | G | A | 0.785 | 3.19E-03 | -0.226 | 1.86E-01 | -0.086 |
| **conditioning for rs36194177** |  | | | | | | | | |
| rs1448484 | 15 | 28283441 | A | G | 0.165 | 1.21E-02 | 0.163 | 8.50E-02 | 0.125 |
| rs1667392 | 15 | 28533565 | G | C | 0.305 | 1.42E-03 | -0.196 | 1.34E-02 | -0.142 |
| rs36194177 | 15 | 29118784 | G | A | 0.208 | 1.39E-02 | 0.171 | NA | NA |
| rs2636060 | 15 | 29425936 | G | A | 0.785 | 3.19E-03 | -0.226 | 2.48E-01 | -0.082 |
| **conditioning for rs2636060** |  | | | | | | | | |
| rs1448484 | 15 | 28283441 | A | G | 0.165 | 1.21E-02 | 0.163 | 9.99E-02 | 0.138 |
| rs1667392 | 15 | 28533565 | G | C | 0.305 | 1.42E-03 | -0.196 | 1.11E-02 | -0.145 |
| rs36194177 | 15 | 29118784 | G | A | 0.208 | 1.39E-02 | 0.171 | 1.62E-01 | 0.094 |
| rs2636060 | 15 | 29425936 | G | A | 0.785 | 3.19E-03 | -0.226 | NA | NA |

**Table S4.** **Omnibus test in the Cape Verde sample, based on haplotypes**

**from our lead signals (rs1448484, rs1667392, rs36194177 and rs2636060).**

df = degrees of freedom.

| **Haplotype** | **Frequency** | **Beta** |
| --- | --- | --- |
| GGAG | 0.22 | Reference |
| AGAG | 0.069 | - 0.039 |
| GGGG | 0.074 | 0.002 |
| AGGG | 0.062 | - 0.229 |
| ACGG | 0.015 | - 0.029 |
| GGAA | 0.076 | - 0.088 |
| AGAA | 0.039 | - 0.144 |
| GGGA | 0.041 | - 0.326 |
| AGGA | 0.227 | - 0.268 |
| ACGA | 0.158 | - 0.466 |
| **R-squared** | 0.063 |  |
| **F-statistic** | 5.03 |  |
| **df** | 9 |  |
| **p-value** | 1.38 x 10^-6^ |  |

**Table S5. Omnibus test in the Cuba sample, based on haplotypes**

**from our lead signals (rs1448484, rs1667392, rs36194177 and**

**rs2636060).** df = degrees of freedom.

| **Haplotype** | **Frequency** | **Beta** |
| --- | --- | --- |
| GGAG | 0.739 | Reference |
| AGAG | 0.018 | 0.127 |
| GGGG | 0.355 | - 0.195 |
| AGGG | 0.028 | - 0.330 |
| GGAA | 0.024 | 0.136 |
| AGAA | 0.067 | - 0.196 |
| ACAA | 0.011 | 0.042 |
| GGGA | 0.028 | - 0.202 |
| AGGA | 0.451 | - 0.106 |
| ACGA | 0.248 | - 0.322 |
| **R-squared** | 0.027 |  |
| **F-statistic** | 2.34 |  |
| **df** | 9 |  |
| **p-value** | 0.013 |  |

**Table S6.** **Follow-up results of all genome-wide signals identified from our meta-analysis in an East African sample [10].**

Chr: chromosome; EA: effect allele; MAF: minor allele frequency; SE: standard error; EAF: East Africa.

| **SNP** | **Chr** | **Position (GRCg37/hg19)** | **EA** | **MAF** | **P-value** | **Beta** | **SE** | **Effect (EAF-Meta)** |
| --- | --- | --- | --- | --- | --- | --- | --- | --- |
| [rs10521009](http://www.ncbi.nlm.nih.gov/SNP/snp_ref.cgi?type=rs&rs=rs10521009) | 5 | 33894764 | A | 0.232 | 0.110 | -0.728 | 0.456 | - - |
| [rs1030241](http://www.ncbi.nlm.nih.gov/SNP/snp_ref.cgi?type=rs&rs=rs1030241) | 5 | 33913369 | C | 0.208 | 0.098 | -0.794 | 0.480 | - - |
| [rs2113111](http://www.ncbi.nlm.nih.gov/SNP/snp_ref.cgi?type=rs&rs=rs2113111) | 5 | 33917250 | T | 0.209 | 0.094 | -0.801 | 0.479 | - - |
| [rs7713279](http://www.ncbi.nlm.nih.gov/SNP/snp_ref.cgi?type=rs&rs=rs7713279) | 5 | 33928902 | T | 0.299 | 0.065 | -0.789 | 0.427 | - - |
| [rs35407](http://www.ncbi.nlm.nih.gov/SNP/snp_ref.cgi?type=rs&rs=rs35407) | 5 | 33946571 | G | 0.338 | 0.279 | -0.455 | 0.420 | - - |
| [rs35395](http://www.ncbi.nlm.nih.gov/SNP/snp_ref.cgi?type=rs&rs=rs35395) | 5 | 33948589 | C | 0.247 | 0.260 | -0.515 | 0.457 | - - |
| [rs35397](http://www.ncbi.nlm.nih.gov/SNP/snp_ref.cgi?type=rs&rs=rs35397) | 5 | 33951116 | T | 0.068 | 0.604 | -0.403 | 0.779 | - - |
| [rs16891982](http://www.ncbi.nlm.nih.gov/SNP/snp_ref.cgi?type=rs&rs=rs16891982) | 5 | 33951693 | G | 0.027 | 0.162 | -1.678 | 1.2 | - - |
| [rs185146](http://www.ncbi.nlm.nih.gov/SNP/snp_ref.cgi?type=rs&rs=rs185146) | 5 | 33952106 | T | 0.247 | 0.298 | -0.478 | 0.459 | - - |
| [rs173662](http://www.ncbi.nlm.nih.gov/SNP/snp_ref.cgi?type=rs&rs=rs173662) | 5 | 33952812 | C | 0.214 | 0.327 | -0.468 | 0.478 | - - |
| [rs35389](http://www.ncbi.nlm.nih.gov/SNP/snp_ref.cgi?type=rs&rs=rs35389) | 5 | 33954880 | A | 0.211 | 0.419 | -0.386 | 0.478 | - - |
| [rs28777](http://www.ncbi.nlm.nih.gov/SNP/snp_ref.cgi?type=rs&rs=rs28777) | 5 | 33958959 | A | 0.215 | 0.309 | -0.484 | 0.476 | - - |
| [rs183671](http://www.ncbi.nlm.nih.gov/SNP/snp_ref.cgi?type=rs&rs=rs183671) | 5 | 33964210 | G | 0.201 | 0.827 | -0.103 | 0.476 | - - |
| [rs11021131](http://www.ncbi.nlm.nih.gov/SNP/snp_ref.cgi?type=rs&rs=rs11021131) | 11 | 88472034 | C | 0.016 | 0.011 | -3.957 | 1.566 | - - |
| [rs12271760](http://www.ncbi.nlm.nih.gov/SNP/snp_ref.cgi?type=rs&rs=rs12271760) | 11 | 88472271 | A | 0.016 | 0.011 | -3.957 | 1.566 | - - |
| [rs35790407](http://www.ncbi.nlm.nih.gov/SNP/snp_ref.cgi?type=rs&rs=rs35790407) | 11 | 88480235 | A | 0.011 | 0.003 | -5.501 | 1.901 | - - |
| [rs4753182](http://www.ncbi.nlm.nih.gov/SNP/snp_ref.cgi?type=rs&rs=rs4753182) | 11 | 88485300 | A | 0.017 | 0.037 | -3.084 | 1.482 | - - |
| [rs4753184](http://www.ncbi.nlm.nih.gov/SNP/snp_ref.cgi?type=rs&rs=rs4753184) | 11 | 88493373 | A | 0.017 | 0.037 | -3.084 | 1.482 | - - |
| [rs2201930](http://www.ncbi.nlm.nih.gov/SNP/snp_ref.cgi?type=rs&rs=rs2201930) | 11 | 88494876 | C | 0.233 | 0.707 | -0.177 | 0.473 | - - |
| [rs12275597](http://www.ncbi.nlm.nih.gov/SNP/snp_ref.cgi?type=rs&rs=rs12275597) | 11 | 88505080 | G | 0.016 | 0.036 | -3.149 | 1.504 | - - |
| [rs11021284](http://www.ncbi.nlm.nih.gov/SNP/snp_ref.cgi?type=rs&rs=rs11021284) | 11 | 88506594 | A | 0.016 | 0.036 | -3.149 | 1.504 | - - |
| [rs1499189](http://www.ncbi.nlm.nih.gov/SNP/snp_ref.cgi?type=rs&rs=rs1499189) | 11 | 88515738 | T | 0.016 | 0.036 | -3.149 | 1.504 | - - |
| [rs11021331](http://www.ncbi.nlm.nih.gov/SNP/snp_ref.cgi?type=rs&rs=rs11021331) | 11 | 88520835 | G | 0.016 | 0.036 | -3.149 | 1.504 | - - |
| [rs3862368](http://www.ncbi.nlm.nih.gov/SNP/snp_ref.cgi?type=rs&rs=rs3862368) | 11 | 88529474 | A | 0.016 | 0.036 | -3.149 | 1.504 | - - |
| [rs12797798](http://www.ncbi.nlm.nih.gov/SNP/snp_ref.cgi?type=rs&rs=rs12797798) | 11 | 88530555 | A | 0.016 | 0.036 | -3.149 | 1.504 | - - |
| [rs4094125](http://www.ncbi.nlm.nih.gov/SNP/snp_ref.cgi?type=rs&rs=rs4094125) | 11 | 88535959 | A | 0.277 | 0.833 | -0.095 | 0.453 | - - |
| [rs12801588](http://www.ncbi.nlm.nih.gov/SNP/snp_ref.cgi?type=rs&rs=rs12801588) | 11 | 88548272 | G | 0.018 | 0.040 | -2.947 | 1.436 | - - |
| [rs3956251](http://www.ncbi.nlm.nih.gov/SNP/snp_ref.cgi?type=rs&rs=rs3956251) | 11 | 88548848 | C | 0.287 | 0.855 | -0.080 | 0.443 | - - |
| [rs6483481](http://www.ncbi.nlm.nih.gov/SNP/snp_ref.cgi?type=rs&rs=rs6483481) | 11 | 88549245 | A | 0.288 | 0.886 | -0.063 | 0.444 | - - |
| [rs11021438](http://www.ncbi.nlm.nih.gov/SNP/snp_ref.cgi?type=rs&rs=rs11021438) | 11 | 88549978 | C | 0.018 | 0.040 | -2.947 | 1.436 | - - |
| [rs12279922](http://www.ncbi.nlm.nih.gov/SNP/snp_ref.cgi?type=rs&rs=rs12279922) | 11 | 88551984 | C | 0.018 | 0.040 | -2.947 | 1.436 | - - |
| [rs11021475](http://www.ncbi.nlm.nih.gov/SNP/snp_ref.cgi?type=rs&rs=rs11021475) | 11 | 88559928 | C | 0.228 | 0.620 | -0.237 | 0.480 | - - |
| [rs492312](http://www.ncbi.nlm.nih.gov/SNP/snp_ref.cgi?type=rs&rs=rs492312) | 11 | 88568379 | G | 0.288 | 0.810 | -0.107 | 0.447 | - - |
| [rs586927](http://www.ncbi.nlm.nih.gov/SNP/snp_ref.cgi?type=rs&rs=rs586927) | 11 | 88571504 | T | 0.283 | 0.826 | -0.099 | 0.451 | - - |
| [rs10831517](http://www.ncbi.nlm.nih.gov/SNP/snp_ref.cgi?type=rs&rs=rs10831517) | 11 | 88574834 | A | 0.017 | 0.013 | -3.773 | 1.517 | - - |
| [rs643566](http://www.ncbi.nlm.nih.gov/SNP/snp_ref.cgi?type=rs&rs=rs643566) | 11 | 88575037 | A | 0.176 | 0.488 | -0.372 | 0.537 | - - |
| [rs645327](http://www.ncbi.nlm.nih.gov/SNP/snp_ref.cgi?type=rs&rs=rs645327) | 11 | 88588786 | C | 0.156 | 0.572 | -0.311 | 0.553 | - - |
| [rs61902997](http://www.ncbi.nlm.nih.gov/SNP/snp_ref.cgi?type=rs&rs=rs61902997) | 11 | 88591918 | C | 0.016 | 0.027 | -3.48 | 1.573 | - - |
| [rs652659](http://www.ncbi.nlm.nih.gov/SNP/snp_ref.cgi?type=rs&rs=rs652659) | 11 | 88599826 | A | 0.212 | 0.987 | -0.007 | 0.495 | - - |
| [rs568109](http://www.ncbi.nlm.nih.gov/SNP/snp_ref.cgi?type=rs&rs=rs568109) | 11 | 88599914 | T | 0.212 | 0.987 | -0.007 | 0.495 | - - |
| [rs11021644](http://www.ncbi.nlm.nih.gov/SNP/snp_ref.cgi?type=rs&rs=rs11021644) | 11 | 88608787 | C | 0.021 | 0.028 | -2.938 | 1.338 | - - |
| [rs10160510](http://www.ncbi.nlm.nih.gov/SNP/snp_ref.cgi?type=rs&rs=rs10160510) | 11 | 88614324 | T | 0.012 | 0.346 | -1.681 | 1.784 | - - |
| [rs632604](http://www.ncbi.nlm.nih.gov/SNP/snp_ref.cgi?type=rs&rs=rs632604) | 11 | 88615165 | C | 0.173 | 0.841 | -0.107 | 0.539 | - - |
| [rs682290](http://www.ncbi.nlm.nih.gov/SNP/snp_ref.cgi?type=rs&rs=rs682290) | 11 | 88615667 | C | 0.221 | 0.745 | 0.157 | 0.483 | + - |
| [rs589675](http://www.ncbi.nlm.nih.gov/SNP/snp_ref.cgi?type=rs&rs=rs589675) | 11 | 88616758 | A | 0.213 | 0.904 | 0.058 | 0.489 | + - |
| [rs10437581](http://www.ncbi.nlm.nih.gov/SNP/snp_ref.cgi?type=rs&rs=rs10437581) | 11 | 88616875 | A | 0.017 | 0.031 | -3.1 | 1.444 | - - |
| [rs655869](http://www.ncbi.nlm.nih.gov/SNP/snp_ref.cgi?type=rs&rs=rs655869) | 11 | 88617288 | A | 0.220 | 0.702 | 0.184 | 0.482 | + - |
| [rs591893](http://www.ncbi.nlm.nih.gov/SNP/snp_ref.cgi?type=rs&rs=rs591893) | 11 | 88617341 | C | 0.220 | 0.702 | 0.184 | 0.482 | + - |
| [rs11018488](http://www.ncbi.nlm.nih.gov/SNP/snp_ref.cgi?type=rs&rs=rs11018488) | 11 | 88861590 | T | 0.018 | 0.246 | -1.724 | 1.489 | - - |
| [rs3097449](http://www.ncbi.nlm.nih.gov/SNP/snp_ref.cgi?type=rs&rs=rs3097449) | 15 | 27857893 | C | 0.426 | 0.484 | 0.274 | 0.392 | + - |
| [rs3101587](http://www.ncbi.nlm.nih.gov/SNP/snp_ref.cgi?type=rs&rs=rs3101587) | 15 | 27858084 | C | 0.436 | 0.437 | 0.303 | 0.391 | + - |
| [rs3101585](http://www.ncbi.nlm.nih.gov/SNP/snp_ref.cgi?type=rs&rs=rs3101585) | 15 | 27858287 | T | 0.435 | 0.457 | 0.291 | 0.391 | + - |
| [rs3098575](http://www.ncbi.nlm.nih.gov/SNP/snp_ref.cgi?type=rs&rs=rs3098575) | 15 | 27858335 | A | 0.436 | 0.437 | 0.303 | 0.391 | + - |
| [rs3098576](http://www.ncbi.nlm.nih.gov/SNP/snp_ref.cgi?type=rs&rs=rs3098576) | 15 | 27858408 | C | 0.436 | 0.437 | 0.303 | 0.391 | + - |
| [rs3097447](http://www.ncbi.nlm.nih.gov/SNP/snp_ref.cgi?type=rs&rs=rs3097447) | 15 | 27859712 | C | 0.430 | 0.494 | 0.267 | 0.391 | + - |
| [rs3097445](http://www.ncbi.nlm.nih.gov/SNP/snp_ref.cgi?type=rs&rs=rs3097445) | 15 | 27860481 | G | 0.430 | 0.494 | 0.267 | 0.391 | + - |
| [rs3101690](http://www.ncbi.nlm.nih.gov/SNP/snp_ref.cgi?type=rs&rs=rs3101690) | 15 | 27861143 | A | 0.430 | 0.494 | 0.267 | 0.391 | + - |
| [rs3097444](http://www.ncbi.nlm.nih.gov/SNP/snp_ref.cgi?type=rs&rs=rs3097444) | 15 | 27863278 | C | 0.425 | 0.624 | 0.191 | 0.390 | + - |
| [rs3101689](http://www.ncbi.nlm.nih.gov/SNP/snp_ref.cgi?type=rs&rs=rs3101689) | 15 | 27863710 | C | 0.419 | 0.756 | 0.121 | 0.392 | + - |
| [rs3097442](http://www.ncbi.nlm.nih.gov/SNP/snp_ref.cgi?type=rs&rs=rs3097442) | 15 | 27865597 | A | 0.399 | 0.744 | 0.128 | 0.394 | + - |
| [rs1448484](http://www.ncbi.nlm.nih.gov/SNP/snp_ref.cgi?type=rs&rs=rs1448484) | 15 | 28283441 | A | 0.363 | 4.43 x 10^-4^ | -1.467 | 0.416 | - - |
| [rs73377768](http://www.ncbi.nlm.nih.gov/SNP/snp_ref.cgi?type=rs&rs=rs73377768) | 15 | 28314426 | A | 0.326 | 0.480 | -0.302 | 0.428 | - - |
| [rs6497263](http://www.ncbi.nlm.nih.gov/SNP/snp_ref.cgi?type=rs&rs=rs6497263) | 15 | 28320661 | T | 0.328 | 0.527 | -0.270 | 0.428 | - - |
| [rs12912427](http://www.ncbi.nlm.nih.gov/SNP/snp_ref.cgi?type=rs&rs=rs12912427) | 15 | 28495956 | G | 0.039 | 0.284 | -1.053 | 0.983 | - - |
| [rs4035940](http://www.ncbi.nlm.nih.gov/SNP/snp_ref.cgi?type=rs&rs=rs4035940) | 15 | 28604617 | G | 0.080 | 0.089 | -1.253 | 0.737 | - - |
| [rs142415892](http://www.ncbi.nlm.nih.gov/SNP/snp_ref.cgi?type=rs&rs=rs142415892) | 15 | 29119211 | A | 0.396 | 0.063 | -0.737 | 0.396 | - - |
| [rs148942115](http://www.ncbi.nlm.nih.gov/SNP/snp_ref.cgi?type=rs&rs=rs148942115) | 15 | 29119964 | C | 0.393 | 0.049 | -0.783 | 0.399 | - - |
| [rs150641715](http://www.ncbi.nlm.nih.gov/SNP/snp_ref.cgi?type=rs&rs=rs150641715) | 15 | 29151348 | A | 0.467 | 0.017 | -0.954 | 0.4 | - - |
| [rs2672699](http://www.ncbi.nlm.nih.gov/SNP/snp_ref.cgi?type=rs&rs=rs2672699) | 15 | 29424902 | A | 0.340 | 0.294 | -0.425 | 0.405 | - - |
| [rs2636060](http://www.ncbi.nlm.nih.gov/SNP/snp_ref.cgi?type=rs&rs=rs2636060) | 15 | 29425936 | A | 0.390 | 0.322 | -0.392 | 0.396 | - - |
| [rs1834640](http://www.ncbi.nlm.nih.gov/SNP/snp_ref.cgi?type=rs&rs=rs1834640) | 15 | 48392165 | A | 0.245 | 1.11 x 10^-60^ | -7.681 | 0.447 | - - |
| [rs2675345](http://www.ncbi.nlm.nih.gov/SNP/snp_ref.cgi?type=rs&rs=rs2675345) | 15 | 48400199 | A | 0.247 | 3.65 x 10^-60^ | -7.718 | 0.451 | - - |
| [rs1426654](http://www.ncbi.nlm.nih.gov/SNP/snp_ref.cgi?type=rs&rs=rs1426654) | 15 | 48426484 | A | 0.243 | 6.89 x 10^-61^ | -7.775 | 0.451 | - - |
| [rs2470102](http://www.ncbi.nlm.nih.gov/SNP/snp_ref.cgi?type=rs&rs=rs2470102) | 15 | 48433494 | A | 0.246 | 1.29 x 10^-59^ | -7.721 | 0.453 | - - |
| [rs8028919](http://www.ncbi.nlm.nih.gov/SNP/snp_ref.cgi?type=rs&rs=rs8028919) | 15 | 48460188 | A | 0.367 | 1.55 x 10^-29^ | -4.863 | 0.422 | - - |
| [rs11070628](http://www.ncbi.nlm.nih.gov/SNP/snp_ref.cgi?type=rs&rs=rs11070628) | 15 | 48471615 | C | 0.431 | 8.69 x 10^-21^ | -4.018 | 0.423 | - - |
| [rs2413887](http://www.ncbi.nlm.nih.gov/SNP/snp_ref.cgi?type=rs&rs=rs2413887) | 15 | 48485926 | C | 0.243 | 1.69 x 10^-60^ | -7.736 | 0.451 | - - |
| [rs10416746](http://www.ncbi.nlm.nih.gov/SNP/snp_ref.cgi?type=rs&rs=rs10416746) | 19 | 3563982 | A | 0.167 | 1.53 x 10^-4^ | 2.019 | 0.531 | ++ |
| [rs112332856](http://www.ncbi.nlm.nih.gov/SNP/snp_ref.cgi?type=rs&rs=rs112332856) | 19 | 3565599 | C | 0.496 | 1.15 x 10^-15^ | 3.344 | 0.413 | ++ |

**Table S7. Follow-up of tanning response signals [14] in our meta-analysis.** Chr: chromosome; EA: effect allele. TR: tanning response data .

*Reported *P*-values (Fixed effects model) were obtained after conditioning for the *SLC24A5* and *SLC45A2* signals, except for rs16891982. ^#^*P*-value based on the initial meta-analysis from our study.

| **SNP** | **Chr** | **Position (GRCg37/hg19)** | **EA** | **Odds Ratio (TR)** | **UKBB**  ***P*-value (TR)** | **Meta-analysis *P*-value (TR)** | **Gene** | ***P*-value meta*** | **Beta meta** | **SE meta** |
| --- | --- | --- | --- | --- | --- | --- | --- | --- | --- | --- |
| rs1308048 | 1 | 66888542 | C | 0.93 | 2.09E-14 | 2.83E-08 | *PDE4B* | 8.92E-01 | 0.00 | 0.03 |
| rs12078075 | 1 | 205163798 | G | 1.09 | 3.99E-09 | 1.71E-04 | *RIPK5* | 7.97E-01 | -0.03 | 0.13 |
| rs9818780 | 3 | 156492758 | C | 1.05 | 3.42E-08 | 1.10E-05 | *PA2G4P4* | 6.04E-01 | -0.02 | 0.03 |
| rs16891982 | 5 | 33951693 | C | 0.4 | 2.02E-176 | 2.45E-36 | *SLC45A2* | 2.13E-23^#^ | -0.32 | 0.03 |
| rs251464 | 5 | 149196234 | C | 0.94 | 2.16E-09 | 2.79E-08 | *PPARGC1B* | 5.82E-01 | 0.02 | 0.03 |
| rs12203592 | 6 | 396321 | T | 1.74 | 1.05E-581 | 4.40E-157 | *IRF4* | 1.40E-03 | -0.21 | 0.07 |
| rs117132860 | 7 | 17134708 | A | 1.3 | 7.63E-23 | 2.93E-04 | *AHR/AGR3* | NA | NA | NA |
| rs2737212 | 8 | 116621214 | C | 1.09 | 4.33E-25 | 4.80E-09 | *TRPS1* | 5.14E-01 | 0.02 | 0.04 |
| rs1326797 | 9 | 12716762 | T | 0.93 | 1.24E-17 | 2.62E-11 | *TYRP1* | 8.37E-01 | 0.01 | 0.04 |
| rs10810650 | 9 | 16873551 | C | 0.87 | 2.38E-59 | 2.10E-29 | *BNC2* | 4.48E-01 | 0.03 | 0.03 |
| **rs35563099** | **10** | **119572403** | **T** | 0.89 | **6.61E-24** | **5.33E-07** | ***EMX2*** | **2.25E-03** | **0.13** | **0.04** |
| rs72917317 | 11 | 68817441 | G | 1.18 | 1.02E-29 | 1.31E-08 | *TPCN2* | 4.87E-04 | -0.30 | 0.09 |
| rs1126809 | 11 | 89017961 | A | 1.29 | 2.42E-172 | 2.59E-75 | *TYR* | 1.73E-03 | -0.15 | 0.05 |
| rs9561570 | 13 | 95156198 | T | 1.06 | 1.41E-09 | 2.95E-07 | *DCT* | 1.51E-01 | -0.05 | 0.03 |
| rs1046793 | 13 | 113539894 | C | 0.93 | 2E-18 | 1.97E-05 | *ATP11A* | 5.83E-01 | -0.02 | 0.03 |
| rs746586 | 14 | 92775967 | T | 1.06 | 6.95E-13 | 1.17E-05 | *SLC24A4* | 6.99E-01 | 0.02 | 0.04 |
| rs12913832 | 15 | 28365618 | A | 0.74 | 6.32E-184 | 2.99E-48 | *HERC2/OCA2* | 3.18E-08 | -0.21 | 0.04 |
| rs369230 | 16 | 89645437 | G | 1.6 | 1E-522 | 8.28E-132 | *MC1R* | 4.54E-01 | 0.03 | 0.03 |
| rs6059655 | 20 | 32665748 | A | 1.69 | 1.44E-315 | 2.98E-99 | *RALY/ASIP* | 1.58E-01 | 0.30 | 0.21 |
| rs11703668 | 22 | 45630335 | G | 0.93 | 1E-16 | 4.42E-04 | *KIAA0930* | 1.80E-01 | -0.05 | 0.03 |

**Table S8. Follow-up of skin pigmentation signals identified in a recent GWAS in a large Latin American sample [9] in our meta-analysis.**

*Reported *P*-values (Fixed effects model) were obtained after conditioning for the *SLC24A5* and *SLC45A2* signals, except for rs16891982 and rs1426654. ^#^*P*-value based on the initial meta-analysis from our study.

| **Skin pigmentation GWAS in Latin Americans** | | | | | | **This study (meta-analysis)** | | |
| --- | --- | --- | --- | --- | --- | --- | --- | --- |
| **SNP** | **Chr** | **Position (GRCg37/hg19)** | **Gene** | **Annotation** | ***P*-value** | ***P*-value*** | **Beta** | **SE** |
| rs3795556 | 1 | 205112911 | *DSTYK* | 3’ UTR | 2.10E-01 | 0.634 | 0.016 | 0.033 |
| rs16891982 | 5 | 33951693 | *SLC45A2* | F374L | 1.30E-117 | 2.13E-23^#^ | -0.317 | 0.032 |
| rs12203592 | 6 | 396321 | *IRF4* | Intronic | 3.20E-10 | 0.001 | -0.208 | 0.065 |
| rs10809826 | 9 | 12682663 | *TYRP1* | Intergenic | 1.10E-03 | 0.735 | -0.012 | 0.036 |
| rs11198112 | 10 | 119564143 | *EMX2* | Intergenic | 1.70E-10 | 0.002 | 0.126 | 0.041 |
| rs7118677 | 11 | 88511524 | *GRM5* | Intronic | 1.10E-09 | 2.33E-05 | -0.133 | 0.032 |
| rs1042602 | 11 | 88911696 | *TYR* | S192Y | 9.10E-10 | 9.20E-10 | -0.227 | 0.037 |
| rs1126809 | 11 | 89017961 | *TYR* | R402Q | 2.50E-09 | 0.002 | -0.153 | 0.049 |
| rs4778219 | 15 | 28213850 | *OCA2* | Intronic | 8.30E-01 | 0.513 | -0.029 | 0.044 |
| rs1800407 | 15 | 28230318 | *OCA2* | R419Q | 6.50E-09 | 0.025 | -0.172 | 0.077 |
| rs1800404 | 15 | 28235773 | *OCA2* | Synonymous/TFB | 5.00E-11 | 9.95E-06 | -0.137 | 0.031 |
| rs12913832 | 15 | 28365618 | *HERC2* | Intronic | 1.00E-17 | 3.18E-08 | -0.206 | 0.037 |
| rs4778249 | 15 | 28380518 | *HERC2* | Intronic | 2.50E-06 | 2.73E-04 | -0.130 | 0.036 |
| rs1426654 | 15 | 48426484 | *SLC24A5* | T111A | 1.60E-130 | 6.32E-39^#^ | 0.403 | 0.031 |
| rs885479 | 16 | 89986154 | *MC1R* | R163Q | 1.90E-07 | 0.340 | -0.073 | 0.077 |
| rs2240751 | 19 | 3548231 | *MFSD12* | Y182H | 1.70E-10 | NA | NA | NA |
| rs17422688 | 20 | 43739119 | *WFDC5* | H97Y | 5.20E-01 | 0.721285 | 0.018 | 0.050 |
| rs5756492 | 22 | 37424991 | *MPST* | Intronic | 4.60E-03 | 0.90425 | 0.004 | 0.035 |
