# Supplementary material for: Meta-analysis of GWA studies provides new insights on the genetic architecture of skin pigmentation in recently admixed populations
Source: BMC Genet. 2019 Jul 17;20:59. doi: 10.1186/s12863-019-0765-5 (PMC6637524; doi:10.1186/s12863-019-0765-5)
Supplement: Supplementary file 4 — Text S1 Supplementary information on the methods followed for the datasets included in the meta-analysis (Cape Verde, SAGE II, GALA II). (DOCX 28 kb) [file 12863_2019_765_MOESM4_ESM.docx]

**Supplementary Text S1**

***Cape Verde dataset***

The Cape Verde dataset consists of individuals from six islands of the Archipelago of Cape Verde [1]. Skin pigmentation was measured with the spectrophotometer DSM II ColorMeter (Cortex Technology, Hadsund, Denmark) on the upper inner arm of each individual, in triplicate. DNA from participants was collected from finger-stick blood, extracted (detailed in [1]), and genotyped using the Illumina Infinium HD Human1M-Duo Deadarray (Illumina, San Diego, California, U.S.A.). After genotyping filters were applied to samples and SNP markers (detailed in [1]), the final dataset consisted of 879,359 SNP markers across 684 unrelated individuals. Haplotype phasing was done with the program SHAPEIT2 [2]. Imputation of non-genotyped SNPs was done at the Sanger Imputation Service, using the Positional Burrows-Wheeler Transform algorithm [3], and the samples of the 1000 Genomes as reference haplotypes. Population structure was evaluated using the software EIGENSOFT [4,5] to perform a principal component analysis (PCA).

***SAGE II Dataset***

The Study of African Americans, Asthma, Genes and Environments (SAGE II) is an ongoing case-control study primarily designed to investigate the genetic and environmental factors of asthma in African American children and adolescents (8 to 21 years old) [6]. In order to be eligible, the four grandparents of the participants had to self-identify themselves as African Americans. Skin pigmentation measurements were taken from 373 individuals in the San Francisco Bay Area, California. Using a DSM II ColorMeter (Cortex Technology, Hadsund, Denmark); pigmentation was measured in triplicate for each individual from the inner side of each upper arm, to obtain a melanin index [7]. DNA samples were genotyped using the Axiom LAT1 array (World Array 4, Affymetrix, Santa Clara, CA, United States). Quality control filters for samples and markers are described elsewhere [6]. The final dataset consists of 797,128 SNPs across 373 unrelated African Americans. Ancestry structure was assessed by PCA using EIGENSOFT [4,5], and imputation of autosomal SNPs was done by means of the Michigan Imputation Server [8] using Minimac3 software [9] after phasing the genotypes with the program SHAPEIT [10], as described elsewhere [7]. The first release of The Haplotype Reference Consortium (HRC) was used as the reference panel [11].

***GALA II Dataset***

The study of Genes-environment and Admixture in Latin Americans (GALA II) is an ongoing case-control study focused on asthma coordinated from the University of California, San Francisco. Recruitment protocols were similar to SAGE II with the only differences being that participants where skin color measurements were obtained were recruited in San Juan, Puerto Rico, and they had four grandparents self-identified as Hispanics/Latinos [12]. Skin color quantification, genotyping, and statistical analyses methods were the same as for SAGE II. The final dataset consists of 746,762 SNPs across 285 unrelated individuals [7,12].

**References**

1. Beleza S, Johnson NA, Candille SI, Absher DM, Coram MA, Lopes J, et al. Genetic Architecture of Skin and Eye Color in an African-European Admixed Population. PLoS Genet. 2013;9.

2. Delaneau O, Zagury JF, Marchini J. Improved whole-chromosome phasing for disease and population genetic studies. Nat Methods. 2013;10:5–6.

3. Durbin R. Efficient haplotype matching and storage using the positional Burrows-Wheeler transform (PBWT). Bioinformatics. 2014;30:1266–72.

4. Price AL, Patterson NJ, Plenge RM, Weinblatt ME, Shadick NA, Reich D. Principal components analysis corrects for stratification in genome-wide association studies. Nat Genet. 2006;38:904–9.

5. Patterson N, Price AL, Reich D. Population structure and eigenanalysis. PLoS Genet. 2006;2:2074–93.

6. White M, Risse-Adams O, Goddard P, Contreras M, Adams J, Hu D, et al. Novel genetic risk factors for asthma in African American children: Precision Medicine and the SAGE II Study. Immunogenetics. 2016;68:391–400.

7. Hernandez-Pacheco N, Flores C, Alonso S, Eng C, Mak ACY, Hunstman S, et al. Identification of a novel locus associated with skin colour in African-admixed populations. Sci Rep. 2017;7:44548.

8. Das S, Forer L, Schönherr S, Sidore C, Locke AE, Kwong A, et al. Next-generation genotype imputation service and methods. Nat Genet. 2016;48:1284–7.

9. Fuchsberger C, Abecasis GR, Hinds DA. Minimac2: Faster genotype imputation. Bioinformatics. 2015;31:782–4.

10. Delaneau O, Coulonges C, Zagury J-F. Shape-IT: new rapid and accurate algorithm for haplotype inference. BMC Bioinformatics. 2008;9:540.

11. McCarthy S, Das S, Kretzschmar W, Delaneau O, Wood AR, Teumer A, et al. A reference panel of 64,976 haplotypes for genotype imputation. Nat Genet. 2016;48:1279–83.

12. Pino-Yanes M, Thakur N, Gignoux CR, Galanter JM, Roth LA, Eng C, et al. Genetic ancestry influences asthma susceptibility and lung function among Latinos. J Allergy Clin Immunol. 2015;135:228–35.
